# Transcriptome-wide analysis of trypanosome mRNA decay reveals complex degradation kinetics and suggests a role for co-transcriptional degradation in determining mRNA levels

**DOI:** 10.1111/mmi.12764

**Published:** 2014-09-15

**Authors:** Abeer Fadda, Mark Ryten, Dorothea Droll, Federico Rojas, Valentin Färber, Jurgen R Haanstra, Clemetine Merce, Barbara M Bakker, Keith Matthews, Christine Clayton

**Affiliations:** 1 Zentrum für Molekulare Biologie der Universität Heidelberg (ZMBH), DKFZ-ZMBH AllianceIm Neuenheimer Feld 282, 69120, Heidelberg, Germany; 2 Flat 1, 172 Gloucester Terrace, London, UK; 3 Centre for Immunity, Infection and Evolution, Institute for Immunology and infection Research, School of Biological Sciences, Ashworth Laboratories, University of EdinburghWest Mains Road, Edinburgh, EH9 3JT, UK; 4 Department of Pediatrics and Systems Biology, Centre for Energy Metabolism and Ageing, University Medical Center Groningen, University of GroningenGroningen, The Netherlands; 5 Department of Molecular Cell Physiology, Faculty of Earth and Life Sciences, VU University AmsterdamAmsterdam, The Netherlands

## Abstract

African trypanosomes are an excellent system for quantitative modelling of post-transcriptional mRNA control. Transcription is constitutive and polycistronic; individual mRNAs are excised by *trans* splicing and polyadenylation. We here measure mRNA decay kinetics in two life cycle stages, bloodstream and procyclic forms, by transcription inhibition and RNASeq. Messenger RNAs with short half-lives tend to show initial fast degradation, followed by a slower phase; they are often stabilized by depletion of the 5′–3′ exoribonuclease XRNA. Many longer-lived mRNAs show initial slow degradation followed by rapid destruction: we suggest that the slow phase reflects gradual deadenylation. Developmentally regulated mRNAs often show regulated decay, and switch their decay pattern. Rates of mRNA decay are good predictors of steady state levels for short mRNAs, but mRNAs longer than 3 kb show unexpectedly low abundances. Modelling shows that variations in splicing and polyadenylation rates can contribute to steady-state mRNA levels, but this is completely dependent on competition between processing and co-transcriptional mRNA precursor destruction.

## Introduction

The rates of transcription, RNA splicing, localization and decay all affect the levels of cellular mRNAs, while protein levels are additionally affected by the rates of translation and protein turnover. In mammalian cells, mRNA abundance makes a contribution of up to 40% to final protein levels (Schwanhäusser *et al.*, 2011). Results from high throughput techniques have revealed that decay rates are critical in overall mRNA homeostasis; genes encoding proteins with similar functions share similar transcript decay rates, and the decay rates respond to external stimuli (Shalem *et al.*, 2009; Munchel *et al.*, 2011; Rabani *et al.*, 2011; Schwanhäusser *et al.*, 2011).

Traditionally, decay rates have been measured by extracting mRNA at various times after transcription shut-off. More recently, a method involving pulse-chase with 4-thiouracil has enabled less invasive measurements, but this method is limited to cell types permissive to the label uptake and incorporation into newly synthesized RNA (e.g. Munchel *et al.*, 2011; Rabani *et al.*, 2011). Independently of the method used, transcriptome-wide decay studies can provide insight into the specificity (Grigull *et al.*, 2004; Mnaimneh *et al.*, 2004; Houalla *et al.*, 2006; Chekanova *et al.*, 2007; Kiss and Andrulis, 2010) and dynamics (Gudipati *et al.*, 2012; Harigaya and Parker, 2012; Schneider *et al.*, 2012; Deneke *et al.*, 2013) of the decay machinery. Some of these studies revealed the presence of feedback loops between synthesis and decay (Shalem *et al.*, 2009; Trcek *et al.*, 2011; Sun *et al.*, 2012; Haimovich *et al.*, 2013). In many cases, decay kinetics are not exponential; this can be explained by models in which the ‘age’ of an mRNA influences its decay rate (Deneke *et al.*, 2013).

African trypanosomes are unicellular parasites with a mode of gene expression that is uniquely suited to the study of post-transcriptional mRNA control mechanisms. Transcription is polycistronic, with no apparent regulation of RNA polymerase II initiation (Palenchar and Bellofatto, 2006; Daniels *et al.*, 2010). Mature mRNAs are *trans* spliced, with addition of a capped spliced leader sequence at the 5′ end (Michaeli, 2011); polyadenylation at the 3′ end is determined by, and coupled to, the *trans* splicing of the next transcript downstream (Michaeli, 2011). Measurements that focused on *trans* splicing of the tubulin mRNA (in an *in vitro* system) (Ullu *et al.*, 1993) and the *PGKB* mRNA (*in vivo*) both found precursor half-lives of about 2 min (Haanstra *et al.*, 2008), which suggests that the mRNA may be spliced once the RNA polymerase is 1–2 kb further downstream (Haanstra *et al.*, 2008). Results for the *HSP70* tandem repeat were consistent with these estimates (Huang and van der Ploeg, 1991). However, it is not known whether this *trans* splicing rate is generally applicable.

Two trypanosome life cycle stages are routinely cultured in the laboratory: the bloodstream form, which is equivalent to the form in mammalian blood, and the procyclic form, equivalent to the form that grows in the midgut of the Tsetse vector. Most open reading frames have more than one possible spliced leader addition site, and often, several polyadenylation sites are used; moreover, the choice of both can vary between life cycle stages (Kolev *et al.*, 2010; Nilsson *et al.*, 2010; Siegel *et al.*, 2010; 2011; Zhang *et al.*, 2010). From RNASeq experiments, approximately 400 genes were judged to be developmentally regulated, with approximately equal numbers being preferentially expressed in each form (Siegel *et al.*, 2010). Numerous studies have shown that developmental regulation of individual mRNAs is associated with differences in their decay rates (Fernández-Moya and Estévez, 2010; Kramer and Carrington, 2011; Clayton, 2013). One example, that of PGKB, has been subjected to quantitative modelling. The *PGKB* mRNA is very unstable in bloodstream forms, and stable in the procyclic form. Measurement and modelling of its synthesis and degradation kinetics indicated that the level of the *PGKB* mRNA was predominantly controlled by the rates of transcription and degradation, and life cycle regulation was achieved by changes in the degradation rate (Haanstra *et al.*, 2008). The extent to which differences in mRNA degradation were responsible for developmental regulation on a transcriptome-wide scale was however unknown.

Degradation of trypanosome mRNAs follows the classic eukaryotic paradigm: the CAF1-NOT complex deadenylates the mRNA (Erben *et al.*, 2014), and this is followed by 5′–3′ degradation by XRNA (Li *et al.*, 2006) or 3′–5′ degradation by the exosome (Clayton and Estevez, 2010). The PAN2/PAN3 deadenylase also has a role in degradation of some mRNAs, and there is, in addition, a deadenylation-independent pathway that is initiated by 5′ degradation (Schwede *et al.*, 2009; Fadda *et al.*, 2013). Studies of mRNA degradation currently rely on transcription inhibition. Unfortunately, pulse-labelling with 4-thio-uracil is not feasible: not only is there hardly any uptake, but there is also a bypass pathway for uridine synthesis (Ali *et al.*, 2013) and a very strong flux of the nucleotide into glycosylation of surface proteins and other pathways (Ali *et al.*, 2012). We previously obtained approximate estimates of bloodstream-form trypanosome mRNA half-lives by inhibiting transcription for 30 min. We demonstrated that for most mRNAs, deadenylation precedes degradation (Manful *et al.*, 2011; Fadda *et al.*, 2013) and also found that the 5′–3′ exoribonuclease XRNA was important mainly for degradation of very unstable mRNAs (Manful *et al.*, 2011). This confirmed previous observations made with individual mRNAs (Schwede *et al.*, 2008; 2009,). The analyses were however limited by the use of a single time-point for transcription inhibition, meaning that half-life estimates were very approximate and complex kinetics could not be detected. Also, procyclic trypanosomes were not studied.

The work described here comprises part of a study that aims to model trypanosome metabolic pathways from genome to metabolome. To allow quantitative modelling, as many parameters as possible must be measured in trypanosomes cultured under tightly defined conditions (Bakker *et al.*, 2010). In this paper we describe a detailed analysis of mRNA decay in both bloodstream form and procyclic trypanosomes, including complex decay kinetics. We find extensive variations in mRNA decay, both between mRNAs and between life cycle stages, but these are usually insufficient to account for steady-state mRNA abundance. By modelling, we uncover a potentially important role for competition between mRNA processing and co-transcriptional RNA destruction in determining steady-state mRNA levels.

## Results

### Developmental regulation of steady-state mRNA levels

In this work we aimed to measure the half-lives of all trypanosome mRNAs in both bloodstream and procyclic forms, and to obtain an overview of *trans* splicing kinetics. To measure mRNA decay we inhibited *trans* splicing and transcription for various times, using Sinefungin to prevent splicing and Actinomycin D to stop transcription; to measure splicing we used Actinomycin D alone. We prepared RNA, depleted rRNA by subtraction and then measured relative RNA amounts with RNAseq. All samples are listed in Supplementary Table S1, which includes the raw data. Supplementary Table S2 summarizes the main results. Details are in subsequent Supplementary Tables. Most analyses concentrate on a non-redundant list of genes in which repeated copies have been removed (Siegel *et al.*, 2010).

We first analysed steady state mRNA levels, comparing results for rRNA-depleted mRNA from four bloodstream form samples and four procyclic samples. Statistically significant differential expression was seen for 693 genes, of which 430 transcripts were more abundant in bloodstream forms (Supplementary Table S3, sheets 1 & 2). We compared the these results with previously published data for poly(A) + mRNA (Siegel *et al.*, 2010; Singh *et al.*, 2014; Vasquez *et al.*, 2014) (Supplementary Figs S1 and S2, and Supplementary Table S3, sheet 3). There were clear differences between the datasets, which could have been caused by differences in the trypanosomes used, the culture conditions, the methods used for RNA preparation and sequencing, and the tools used for data extraction and analysis. This illustrates the fact that for quantitative work, it is vital to control all conditions and methods as tightly as possible.

**Figure 1:**
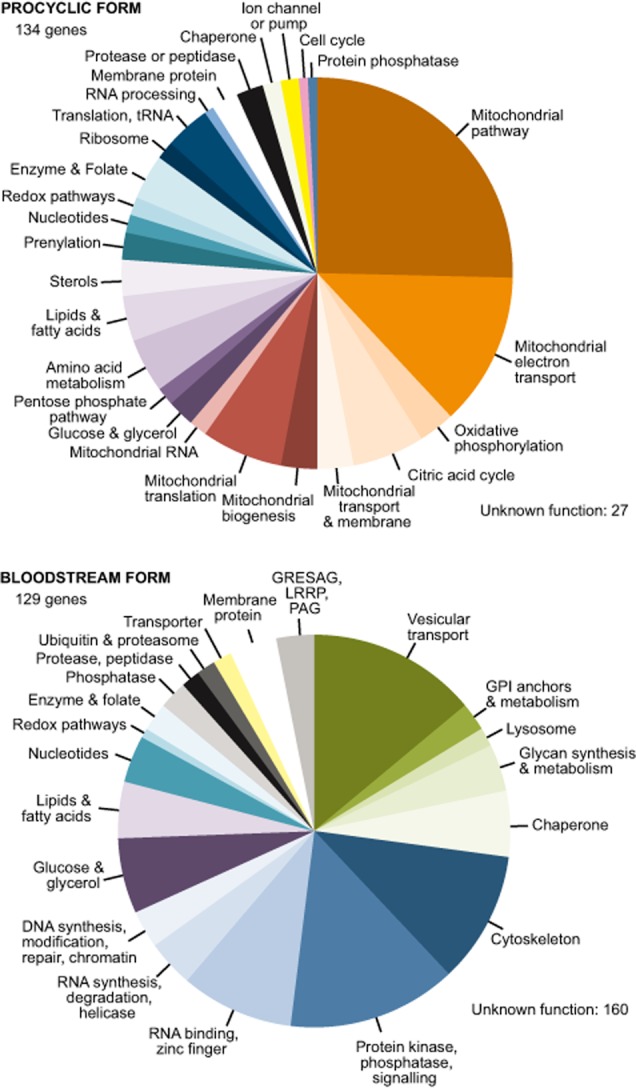
Developmentally regulated mRNAs. For each stage, genes that were upregulated in at least three of the four available RNASeq datasets were selected (Supplementary Table S3, sheet 4) and grouped according to manually assigned functional categories. Genes encoding proteins of no known function (27 for procyclic forms, 160 for bloodstream forms) are not shown. ‘Upregulated’ means at least twofold more abundant than in the other stage; for our current dataset it also means significantly regulated.

The comparisons enabled us to compile a list of the 450 most reproducibly developmentally regulated mRNAs (at least twofold regulated in at least 3 of the 4 available RNA-Seq datasets, Supplementary Table S3, sheet 4). We grouped these according to manually annotated functional categories. Rather surprisingly, almost twice as many mRNAs were reproducibly upregulated in bloodstream forms than were upregulated in procyclics; and oddly, genes with no known function accounted for all of the difference. Figure 1 shows just the upregulated genes that could be functionally assigned. The regulation of energy metabolism was as expected – mitochondrial pathways present in procyclic forms, and reliance on glycolysis in bloodstream forms (Bringaud *et al.*, 2006). Bloodstream forms are also known to have a high requirement for vesicular transport and GPI anchor biosynthesis (Overath and Engstler, 2004). Bloodstream-form-specific expression of genes encoding cytoskeletal proteins, protein kinases and phosphatases, and RNA-binding proteins was less expected. Levels of RNA, however, do not always correlate with levels of protein (Gunasekera *et al.*, 2012; Urbaniak *et al.*, 2013). Translation (Vasquez *et al.*, 2014) and protein degradation are also important.

Our procyclic forms were grown with proline as the principal carbon source, with glucose only from the diluted serum. In contrast, the procyclic forms used by others (Siegel *et al.*, 2010; Vasquez *et al.*, 2014) were grown in medium with 5 mM glucose. Thirteen mRNAs reproducibly showed at least a threefold greater increase in our procyclics [ribo- and poly(A) + datasets] than was seen in the RNASeq data for glucose-grown cells (Supplementary Table S3, sheet 3, columns O–R; names in bold in column C). The increased expression of proline dehydrogenase (Tb927.7.210) has been reported previously (Lamour *et al.*, 2005), and glutamine synthetase (Tb927.7.4970) is next on the same pathway (Bringaud *et al.*, 2006). The mRNAs encoding glycosomal malate dehydrogenase (Tb927.10.15410), ubiquinol-cytochrome *c* reductase (Tb927.4.4990) and an aquaporin (Tb927.6.1520) are also upregulated in proline-cultured cells. Specifically increased mRNAs encoding proteins of unknown function were Tb927.4.3500, Tb927.9.13200, Tb927.11.1690 and Tb927.10.2360. This shows again that carbon source availability affects expression levels in trypanosomes.

### The heterogeneity of *trans* splicing and polyadenylation sites precludes transcriptome-wide rate measurement of processing kinetics

In order to model gene expression, we needed an estimate of the variability in pre-mRNA splicing rates. In an attempt to obtain this, we treated cells with Actinomycin D alone for the shortest feasible time, 5 min, and subjected rRNA-depleted RNA to RNASeq. Since the three previously measured splice times were in the range of 1–2 min, time intervals in that range would have been preferable, but were not technically feasible. For the splicing measurements we used two replicates for bloodstream forms and one for procyclic forms, together with one untreated sample for each. Reads aligning to intergenic regions within a transcription unit come from un-spliced mRNA. *Trans* splicing involves *trans* esterification followed by destruction of the intergenic RNA by exoribonucleases. Most trypanosome mRNAs, however, have multiple possible splice sites (Kolev *et al.*, 2010; Nilsson *et al.*, 2010; Siegel *et al.*, 2010; 2011; Zhang *et al.*, 2010), which means that many RNA sequences can be either intergenic or within an mRNA. We collected all documented splice acceptor sites for both procyclic and bloodstream forms from TriTrypDB, and merged them with our own identified sites. For each site, the read counts in the 20mer upstream region were extracted, normalized to the read counts for the corresponding gene, and the ratio between untreated and Actinomycin D-treated samples was calculated. We then calculated the pre-mRNA half-life for each site, assuming exponential kinetics. If an open reading frame had more than one splice site, we chose the splice site with the shortest half-life to infer splicing kinetics for that open reading frame. 3029 measurements of 5′ splicing for bloodstream forms and 1554 for procyclic forms are shown in Supplementary Table S2 and displayed in Supplementary Fig. S3. The measurements were limited by the numbers of reads, as well as by the use of only a single time interval. The median 5′ pre-mRNA half-life measured in both life stages was a little over 3 min, and many of the measured half-times were between 1–2 min. Since these times are similar to those measured for tubulin, HSP70 and PGK mRNAs, they may be correct. However, there was no correlation in 5′ splicing times between the two life stages even for genes sharing the same major acceptor site in the two stages (not shown). Also, many precursor half-lives were over 4–5 min. Although this could reflect slow splicing, it could also indicate persistence of bicistronic RNAs, or of monocistronic mature mRNAs that had upstream *trans* splice sites. The un-spliced *PGK* precursor is degraded with a half-life of 8 min (Haanstra *et al.*, 2008) so half-lives above this are unlikely to reflect splicing, but may instead reflect precursor degradation. It was thus apparent that for most mRNAs, the complexity of trypanosome mRNA splicing patterns precluded reliable kinetic analysis using the high-throughput method that we had adopted. Our results therefore could not be used to assess the kinetics of splicing. Polyadenylation sites are even more heterogeneous so we made no attempt to analyse 3′-processing kinetics.

Despite the limitations in the results, they did suggest that many trypanosome mRNAs might be spliced within a few minutes of synthesis. Based on this, we adopted a conservative splicing half-time of 2 ± 1.5 min in the modelling (below).

### Trypanosome mRNAs show complex decay kinetics

We next analysed the data for parasites treated with both Sinefungin and Actinomycin D, using multiple time intervals and at least two replicates for each time point. To normalize the data we hybridized Northern blots with a probe complementary to the spliced leader (see Methods and Supplementary Fig. S4). The spliced leader provides a good measure of the number of mRNAs present, because it is present at the 5′-end of every mRNA. Unlike the poly(A) tail, it does not vary in length; and it will be removed by XRNA very fast after the mRNA has been decapped.

**Figure 2:**
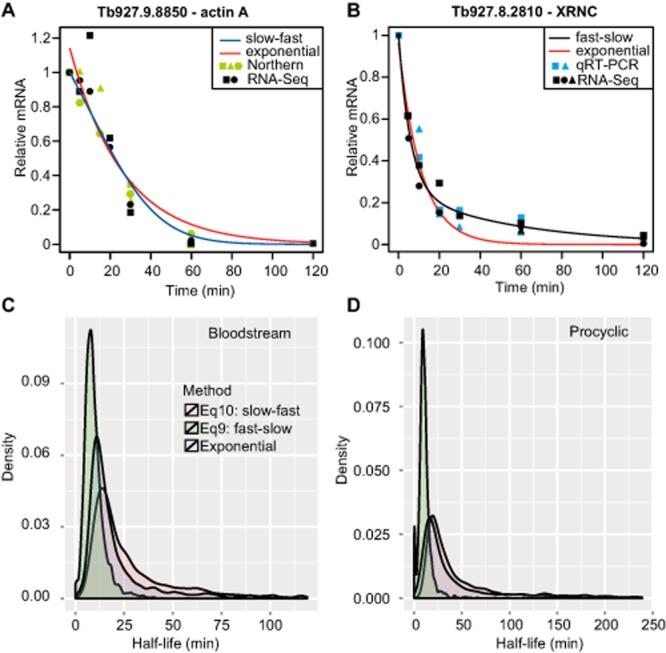
Kinetics of mRNA decay. A. Degradation of the mRNA encoding Actin A in bloodstream forms. RNASeq data are plotted in black, the exponential curve fit is in red, and the slow-fast curve fit is in blue. The slow-fast curve matches better, but is still not ideal. The green points are Northern blotting results (Schwede *et al.*, 2008; 2009,). B. Degradation of the *XRNC* mRNA in bloodstream forms fits the fast-slow model. Details as for (A), except that the cyan points are real-time qRT-PCR data (summarized in Manful *et al.*, 2011). C. The numbers of mRNAs fitting each decay model in bloodstream forms were used to calculate and plot probability density functions for half-live values. The area under the curve represents the probability of having a range of half-life values. Each open reading frame is represented once, with the best-fitted model. D. As for (C), but for procyclic forms.

To calculate half-lives, we considered three possible scenarios: simple exponential decay, or one of two non-exponential patterns (Deneke *et al.*, 2013). The non-exponential models represent a multi-step degradation process in which different mRNA molecules are in different stages of degradation; hence the ‘age’ of the mRNA population (i.e. the length of time after transcription has stopped) plays a role in the shape of overall decay curve (Deneke *et al.*, 2013). In the first scenario, the mRNA population initially seems relatively stable but becomes increasingly unstable over time: we call this the ‘slow-fast’ scenario. For example, the actin mRNA shows little degradation over the first 10 minutes, but then the rate increases (Fig. 2A). In the second scenario, part of the RNA is degraded very rapidly, but a fraction appears to be much more stable (fast-slow scenario). An example of this is shown in Fig. 2B. The model that fit the data best (smallest least-squares error) was used to calculate a single transcript lifetime and half-life for each mRNA and the resulting half-lives (Supplementary Table S4) were used in all further analyses. The results are summarized in Table 1. Sometimes the fit was always poor, especially when there was extensive scatter at early time points (Supplementary Fig. S5A and B), while in other cases, moving from an exponential model to one of the other models clearly gave a better fit (Supplementary Fig. S5C and D). As noted previously (Deneke *et al.*, 2013), transcripts with short half-lives tended to fit the fast-slow model, while those with long half-lives tended to fit a slow-fast model (Fig. 2C and D).

**Table 1:** Summary of half-life results

Category	Bloodstream	Procyclic
Slow-fast	1903	2632
Fast-slow	3074	1622
Exponential	1495	1704
Stable	92	149
Not measured	480	993

Results given are for 6951 unique open reading frames. mRNAs that had half-lives over 120 min (bloodstream forms) or 240 min (procyclic forms) were mostly fitted using the exponential model, but were classified as stable since the half-life could not be measured well using the time points that we had. ‘Not measured’ usually means that the numbers of counts were insufficient to calculate a half-life and/or that the data could not be fitted with any of the three models.

**Figure 3:**
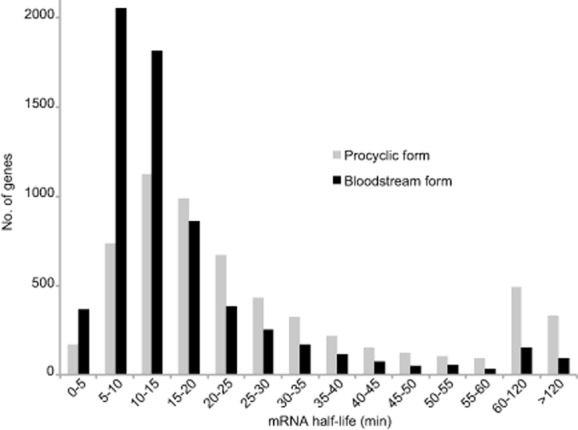
Most trypanosome mRNAs have half-lives of less than 30 min. All mRNAs with measurable half-lives (stable ones are excluded) were placed into classes according to half-life range. The numbers of mRNAs in each class are plotted. All data are for ribo-minus RNA preparations.

Some mRNAs showed no significant degradation over the incubation period, and for some, the abundance was too low for a half-life measurement. However, the use of multiple time points and complex kinetic models enabled us to estimate half-lives for most mRNAs. Bloodstream trypanosome mRNAs had a modal half-life of 9 min, median 12 min, and arithmetic mean 16 min (Fig. 3), similar to the numbers that we previously reported (Manful *et al.*, 2011). Procyclic trypanosomes are larger than bloodstream forms, grow at 27°C, have about twice as many mRNAs per cell and divide more slowly (Haanstra *et al.*, 2008); correspondingly, their mRNAs had a modal half-life of 12 min, median of 20 min, and arithmetic mean of 34 min (Fig. 3, Supplementary Table S3 sheet 5).

### RNASeq half-lives are similar to those found by other methods

We previously had estimated half-lives for bloodstream forms by measuring the amount of mRNA remaining after 30 min of transcription inhibition (Manful *et al.*, 2011). The correlation between our new and old datasets was best for mRNAs with longer half-lives (Supplementary Fig. S6). One likely reason for this is that the reliability of RNASeq decreases at low read densities, and unstable RNAs give very few reads after 30 min of degradation.

**Figure 4:**
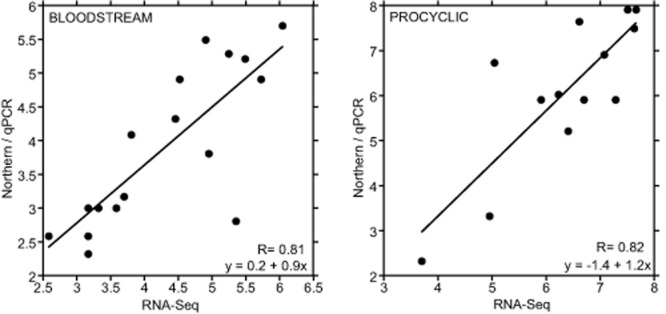
Comparison between RNASeq and other methods. All available RNA half-lives measured by qRT-PCR or Northern blotting were compared with the half-lives measured by RNA-Seq. Both axes show the log (base 2) of the half-life in min. References are in Table 2. If more than one Northern measurement was available the arithmetic mean was used. Only measurements with sufficient time points were included: for example, the numbers designated as ‘more than’ or ‘less than’ a certain half-life in Table 2 were excluded. The formulae and correlation coefficients were calculated with log2-transformed numbers.

The half-lives that we measured by RNA-Seq were in quite good agreement with previously reported half-lives measured by real-time PCR or Northern blotting, with correlation coefficients (for log-transformed data) of over 0.8 (Fig. 2A and B; Fig. 4, Table 2). When we compared the RNASeq results with our previous measurements made using the same protocols for RNA synthesis inhibition, differences in the kinetics were rather minor (Supplementary Fig. S7). RNA-Seq might be somewhat more sensitive at the later time-points, when RNA levels dropped even below the levels that could be measured reliably by q-PCR. Disagreements with literature values (Table 2) could be due to differences in the cell culture conditions as well as in methodology.

**Table 2:** Comparison of RNASeq half-lives with Northern blot results

		Manful BS	BS new	BS Northern/qPCR	PC new	PC Northern	Ref
Not regulated							
Tb927.5.4170	Histone H4	68	45#	30–45	stable	> 60 min	PC (np); BS (Schwede *et al.*, 2009) (Färber *et al.*, 2013)
Tb927.9.8850	Actin A	28	22#	15–25	33#	∼ 90, 52#, 70#	BS e.g. (np), (Hotz *et al.*, 1997); PC (np), (Estévez, 2008; Kramer *et al.*, 2008)
Tb927.1.2330	b-TUB	70	66#	45–60#	135#	∼ 120	(Manful *et al.*, 2011) # (Kramer *et al.*, 2008; Schwede *et al.*, 2008)
Tb927.7.3780	PAP-like	9	9	8	15#	na	(Manful *et al.*, 2011)
Tb927.8.2810	XRNC	11	6	6	9	na	(Manful *et al.*, 2011)
Tb927.4.410	CAF40	26	23#	30	46#	na	(Manful *et al.*, 2011)
Tb927.11.16600	EAP2	9	12	8	5	na	(Manful *et al.*, 2011)
Tb927.11.16310	Myosin	16	31	14	11	na	(Manful *et al.*, 2011)
Tb927.11.4710	Hyp	9	10	8	13	na	(Manful *et al.*, 2011)
Tb927.11.790	Hyp	11	6	6	18	na	(Manful *et al.*, 2011)
Tb927.11.8710	Hyp	10	9	6	9	na	(Manful *et al.*, 2011)
Tb927.6.2810	ABC	11	9	5	16	na	(Manful *et al.*, 2011)
Tb927.7.6980	SF3b	15	14	17	28	na	(Manful *et al.*, 2011)
Tb927.6.4340	LSm5	10	13#	9	15	na	(Manful *et al.*, 2011)
Tb927.11.11330	HSP70	21	38#	39	102	na	(Manful *et al.*, 2011)
Tb927.6.3740	PDI2	63	55	na	156	∼ 60	(Kelly *et al.*, 2012)
Tb927.11.5050	FRm	23	36	na	85#	37	(Estévez, 2008)
Tb927.11.10920	Hyp	11	12	na	31	∼ 10	(Stern *et al.*, 2009)
Tb927.10.12700	PYDHE	53	36	na	stable	45	(Stern *et al.*, 2009)
Bloodstream-specific							
Tb927.10.7090	AOX	stable	stable	stable	na	3.2 h	(Chaudhuri *et al.*, 2002)
Tb927.10.5620	ALD	stable	stable	90#	182	∼ 4 h#	(Mayho *et al.*, 2006)
Tb927.2.6000	GPI-PLC	stable	53#	30#	13	5	(Webb *et al.*, 2005)
Procyclic-specific							
Tb927.10.10250	EP procyclin	na	na	5–10	98	200	(Hotz *et al.*, 1997) and others
Tb927.1.4100	COX IV	14	21#	< 30	216	> 4 h#	(Mayho *et al.*, 2006)
Tb927.9.3170	COXV	15	9#	< 30	203	∼ 4 h	(Mayho *et al.*, 2006)
Tb927.10.280	COX VI	15	11#	< 30	198	∼ 3 h	(Mayho *et al.*, 2006)
Tb927.3.1410	COX VII	26	12	< 30	stable	∼ 6 h#	(Mayho *et al.*, 2006)
Tb927.4.4620	COXVIII	17	5	< 30	stable	∼ 6 h#	(Mayho *et al.*, 2006)
Tb927.10.8320	COX IX	26	14#	< 30	stable	∼ 8 h#	(Mayho *et al.*, 2006)
Tb927.7.7420	ATP synth a	17	22#	< 30	199	> 60#	(Brown *et al.*, 2001)
Tb927.3.1380	ATP synth b	32	27#	< 30	152	> 90#	(Brown *et al.*, 2001)
Tb927.5.1710	ATP synth 9	30	22#	< 30	stable	> 240	(Brown *et al.*, 2001)
Tb927.6.2170	GrpE	38	30#	45	130#	na	(Manful *et al.*, 2011)
Tb927.11.6280	PPDK	36	41	7^*^^*^	stable	na	(Haile *et al.*, 2003)
Tb927.4.4730	AATP11	18	28#	na	104#	82#, 40#	# (Estévez, 2008), (Stern *et al.*, 2009)
Tb927.7.4970	GS	12	10	na	75#	65#	# (Estévez, 2008)
Tb927.10.10770	GCS1	31	29	na	60#	60	(Estévez, 2008)
Tb927.3.4500	FRc	16	38	na	stable	116	(Estévez, 2008)
Tb927.6.1520	AQP3	81	41#	na	stable	∼ 80	(Stern *et al.*, 2009)

Manful BS – Bloodstream-form RNASeq results from (Manful *et al.*, 2011). BS new, PC new: results from the current analysis; BS = bloodstream forms. PC = procyclic forms. BS or PC Northern: Estimates from Northern blots, using data from the references cited on the right hand column. Note that for many of these, estimates of accuracy are not available and the methods for inhibiting transcription varied. All half-lives are in minutes. Non-standard gene abbreviations are: b-TUB – beta tubulin; Myosin HC – myosin heavy chain; Hyp – hypothetic open reading frame encoding protein of unknown function ABC – ABC transporter; PAP – poly(A) polymerase-like; ATP synth – ATP synthase (subunits a-alpha, b-beta, and 9). Values with asterisks are for reporter mRNAs with the 3′-UTR of the gene concerned, synthesized by RNA polymerase I (^*^) or T7 polymerase (^*^^*^). Transcription by T7 polymerase might affect the half-life (Colasante *et al.*, 2007). (np) – our data, not published. #Slow-fast kinetics.

### Changes in mRNA decay partially account for developmental regulation

**Figure 5:**
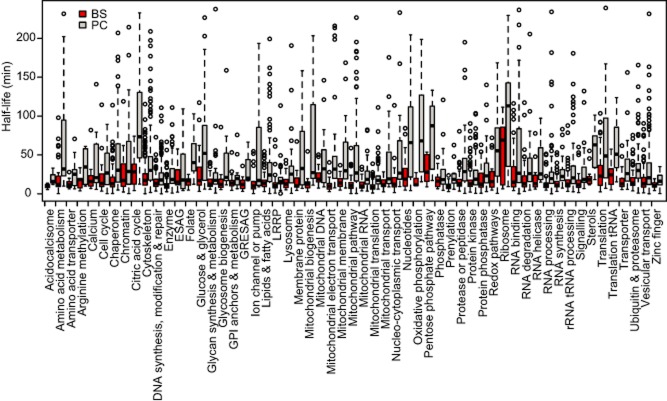
Half-lives of mRNAs in particular functional classes. Unique coding regions were placed into functional classes based on their genome annotation, user comments and publications. The classes used are indicated in Supplementary Table S5. In this box-and-whisker plot the bottom and top of the box are the first and third quartiles, the band inside the box is the median, the whiskers represent the lowest datum still within 1.5 of the lower quartile, and the highest datum still within 1.5 of the upper quartile and the circles represent outliers.

We next asked whether mRNAs encoding proteins with particular functions showed similar half-lives. We grouped mRNAs into functional classes (Supplementary Table S2) and plotted half-lives for each class (Fig. 5). A one-way anova showed significant life-time differences between the different classes (*P*-value << 0.01), suggesting co-ordinate regulation (Supplementary Table S5). Transcripts encoding ribosomal proteins are particularly stable in both stages: large amounts of mRNA are produced from only 1–3 open reading frames (Manful *et al.*, 2011). Although all RNA classes were overall more stable in the procyclic stage (T-test results in Supplementary Table S5), more pronounced shifts were apparent for functions related to procyclic energy metabolism, such as amino acids, citric acid cycle, mitochondrial biogenesis and oxidative phosphorylation. The small number of mRNAs involved in folate and sterol metabolism also showed greater increases in half-life than average. These results were consistent with the idea that developmental regulation is, at least in part, mediated through changes in half-life.

To analyse the relationship further we plotted half-life ratios (bloodstream half-life/procyclic half-life) against the abundance ratios (bloodstream abundance/procyclic abundance). There was almost no correlation, except for very strongly regulated mRNAs (Supplementary Fig. S8). Moreover, not all mRNAs with regulated turnover were regulated in abundance. Restriction of the dataset to the mRNAs that were rapidly spliced in both forms, or using only those mRNAs that had the same splice site in both forms, did not increase the correlation (not shown). However, this analysis almost certainly under-estimated the contribution of mRNA turnover to developmental regulation. We had to exclude not only all stable mRNAs, but also those for which abundance in one stage was too low for the half-life to be measured. The latter included 29 procyclic-form-specific and 172 bloodstream-form-specific mRNAs (Supplementary Table S3, sheet 3). Excluding these, we found 179 mRNAs in which mRNA decay played a clear role in regulation (Supplementary Table S3, sheet 6). We saw regulated mRNA decay not only for components of the cytochrome oxidase (Mayho *et al.*, 2006) and ATP synthase (Brown *et al.*, 2001) complexes (Table 2), but also for 67 other mitochondrial proteins, the AATP11 amino acid transporter, and 9 glycosomal or cytosolic enzymes involved in procyclic energy metabolism. For bloodstream form-specific expression, we detected regulation for the GPI-phospholipase C (Webb *et al.*, 2005); the mRNAs encoding fructose bisphosphate aldolase and the alternative oxidase were relatively stable in procyclic forms but showed no decay at all during our assay in bloodstream forms (Table 2).

Overall, more mRNAs fit a slow-fast model in procyclic than in bloodstream forms (Table 1), and no fewer than 1110 transcripts had different decay models in the two life stages. 976 mRNAs switched from fast-slow in bloodstream forms to slow-fast in procyclics. These were particularly enriched for mitochondrial processes that are developmentally upregulated in procyclics: mitochondrial translation, RNA processing, electron transport, biogenesis and other pathways. Other classes that showed this switch were prenylation, RNA processing and the catch-all ‘enzyme’ group. The 134 that switched the other way were mostly upregulated in bloodstream forms and were enriched for GPI anchors and metabolism, vesicular transport, calcium regulation and DNA synthesis, modification and repair.

### Many mRNAs are less abundant than expected from their half-lives

**Figure 6:**
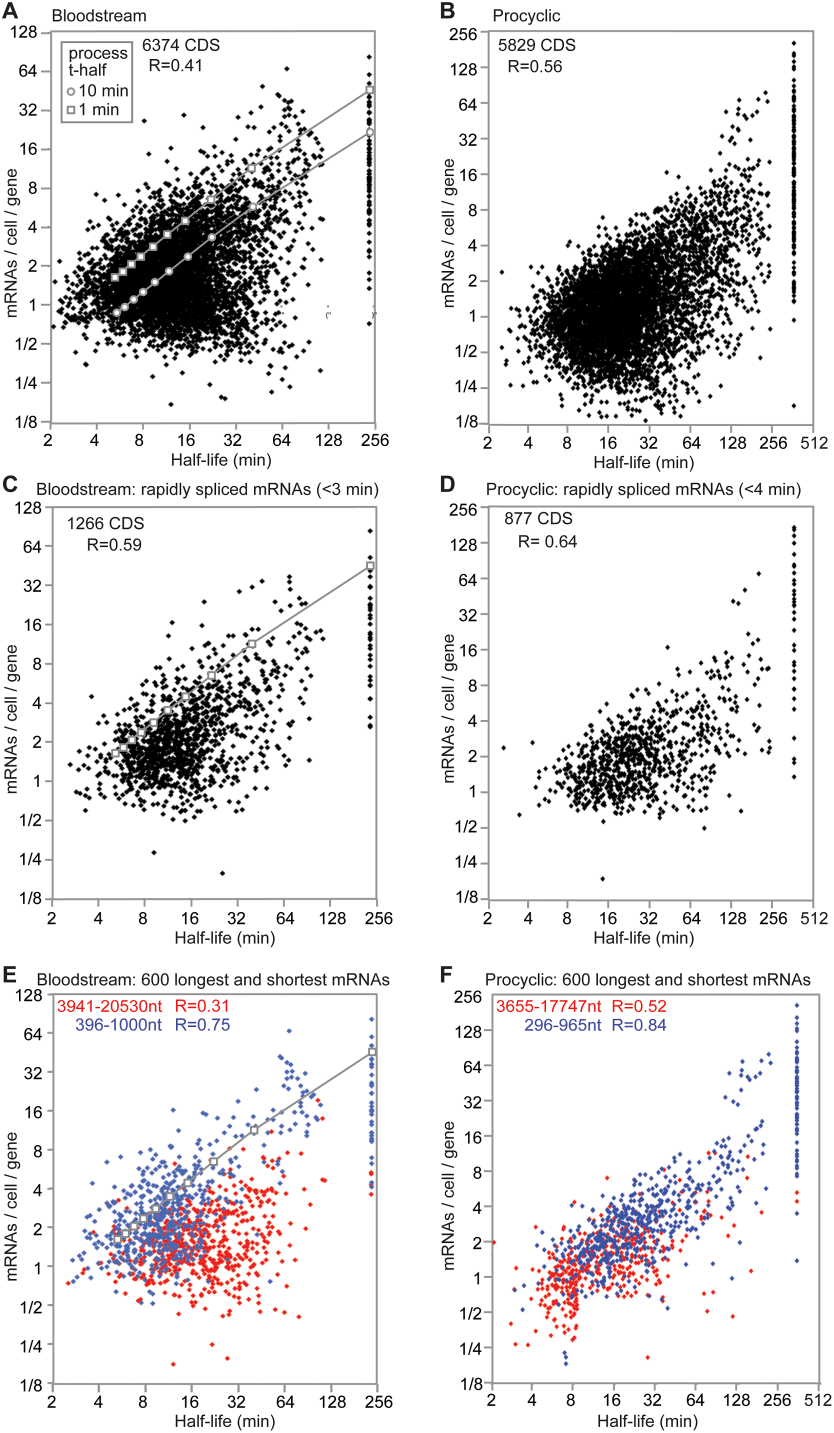
Relationship between half-life and mRNA abundance. A. Relationship between the number of mRNAs per cell per gene and the half-life for 6367 non-redundant open reading frames in bloodstream forms. All mRNAs with apparent half-lives of less than 2 min were excluded since (given the 5 min Sinefungin treatment) this is outside the range of the assay. The mRNAs that appeared stable have been arbitrarily assigned half-lives of 240 min. The Pearson correlation coefficient (R) was calculated from log-transformed data. The grey squares indicate the results of modelling steady-state mRNA abundance in the published model (Haanstra *et al.*, 2008) assuming a splicing half-time of 1 min; the circles are for an assumed 5′-*trans* splicing half-time of 10 min. The gene copy numbers were estimated from random shotgun sequences of genomic DNA. B. As for (A), but for 5831 non-redundant open reading frames in procyclic forms. The mRNAs that appeared stable have been arbitrarily assigned half-lives of 360 min; their median number of transcripts per gene per cell is 9. C. As for (A) but including only mRNAs with precursor half-times of less than 3 min. D. As for (B) but including only mRNAs with precursor half-times of less than 4 min. E. As for (A), but including only the 600 longest (red) and shortest (blue) mRNAs. The length ranges and correlation coefficients are also shown for the two groups. The grey squares indicate the results of modelling steady-state mRNA abundance in the published model (Haanstra *et al.*, 2008) assuming a splicing half-time of 1 min. Lengths were taken from TritrypDB; all mRNAs lacking annotated poly(A) sites were excluded. F. As for (B), but including only the 600 longest (red) and shortest (blue) mRNAs.

Since trypanosome mRNAs are constitutively transcribed, gene copy number and mRNA half-life are expected to make a significant contribution to steady-state mRNA abundance. We therefore normalized mRNA copy numbers per cell to the gene copy number, then compared these values with the half-life. Results suggested that half-life contributes to, but cannot completely account for, the mRNA level (Fig. 6). High abundance was possible only if the mRNA was relatively stable: for example, nearly all mRNAs present at more than 16 copies/cell/gene in bloodstream forms or 32 copies per cell in procyclics had half-lives longer than 30 min.

**Figure 7:**
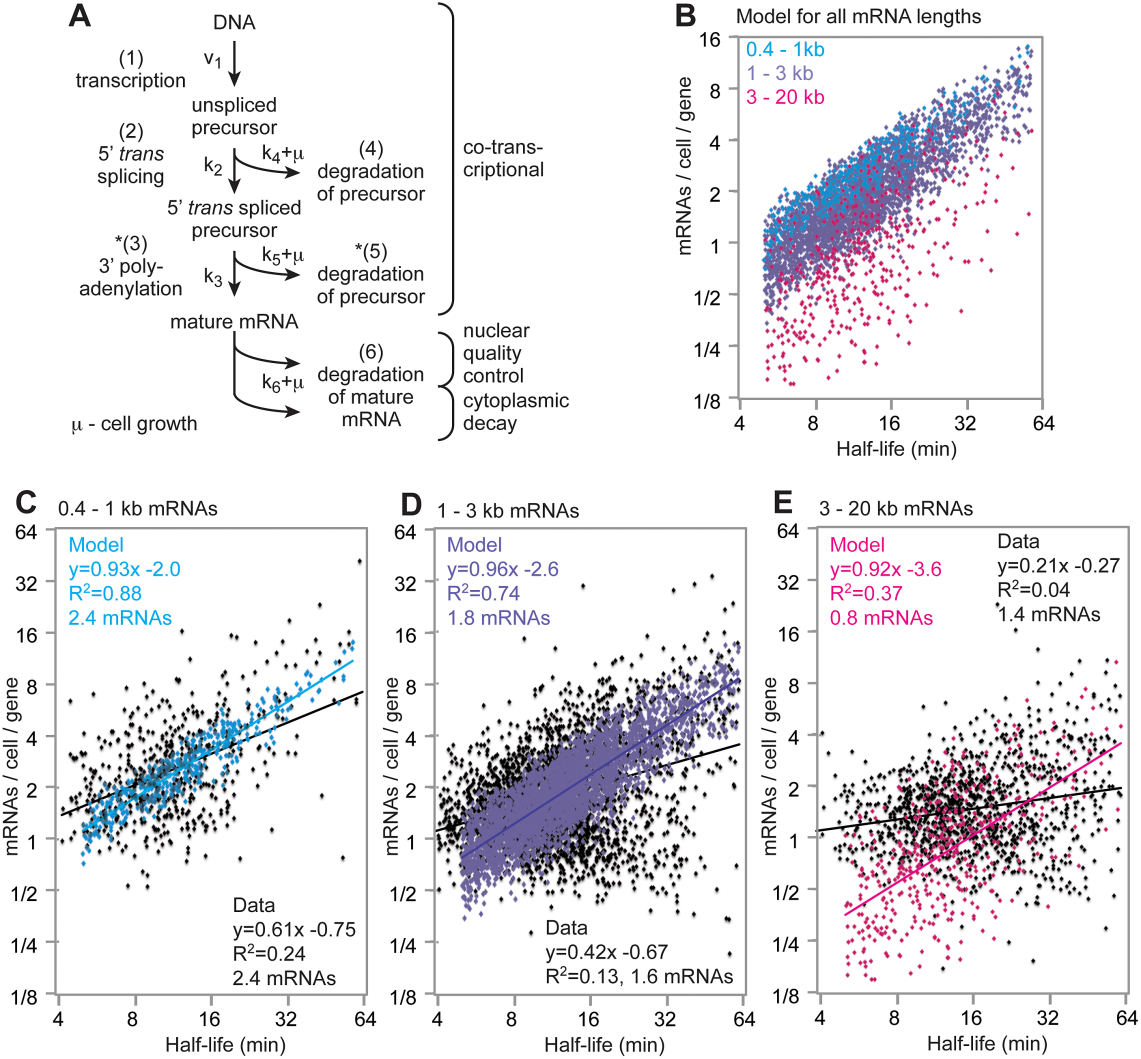
The kinetic model for trypanosome gene expression. A. Cartoon showing the main aspects of the model. *Steps 3 and 5 are extensions compared to (Haanstra *et al.*, 2008). B. Trypanosome mRNA behaviour was modelled for three different groups: 550 mRNAs of 0.4–1, 4408 of 1–3 and 551 of 3–20. This distribution approximates to that seen in our dataset. For each set, half-lives were distributed as in Fig. 4 up to 120′. The amount of mRNA at steady state was then modelled using the following parameters: Transcription rate 0.24 molecules cell^−1^ min^−1^; Splicing and polyadenylation half-lives were sampled from a normal distribution around 2 ± 1.5′. The constant for degradation of the 5′ spliced precursor *k*_5_ (0.08 min^−1^) was multiplied by the ratio (mRNA length in nt)/(600 nt). Shown is the mature mRNA half-life range of 5–60 min, since this includes most transcripts. C. Measured abundances of mRNAs of 0.4–1 kb with half-lives of 5–60 min were plotted over the results from the model. The regression line formulae and correlation coefficients are for log2-transformed data. Median mRNAs/cell/gene are also indicated. D. As (C) but for 1–3 kb mRNAsE. E. As (D) but for mRNAs longer than 3 kb.

We previously developed a model for gene expression in bloodstream trypanosomes, based on detailed measurements for the *PGK* and tubulin genes (Haanstra *et al.*, 2008). We expanded this model in order to account for our new results (Fig. 7A, Table 3). In the published model, the first step is transcription; we had set the overall rate (initiation plus elongation) so that it was sufficient to produce the measured steady state level of mRNA (step 1 in Fig. 7A). The next step (step 2 in Fig. 7A) is 5′ splicing, with a fixed, half-time of 1 min. Splicing competes with precursor degradation rate (step 4 in Fig. 7A); for this, we used a half-life of ∼ 8.7 min, as determined for the *PGK* precursor (Haanstra *et al.*, 2008). Mature mRNA degradation (step 6 in Fig. 7A) includes degradation in both the nucleus and the cytoplasm. The model also includes dilution of RNA species via parasite growth (specific growth rate, μ). To start the new analysis, we used this published model to calculate the expected amounts of mRNA at steady state for various half-lives (grey squares, Fig. 6A). We found that they were towards the upper edge of the cloud of data points. This indicates that the published model predicts almost the maximum amount of mRNA that can be obtained per gene *in vivo*. Notably, however, the levels of many mRNAs fell well below the estimates from the published model. The first, and most obvious, explanation was that we had not allowed for the possibility that splicing rates might vary. We therefore went back to our measurements, and plotted the half-lives and abundances of mRNAs with relatively short measured 5′ precursor half-lives (Fig. 6C and D): although the correlation was marginally better than for the bulk population, this could be an artefact of the lower sample size. Returning to the published model, we plotted the RNA abundances for a 5′ *trans* splicing reaction half-time of 10 min (grey circles, Fig. 6A). This still failed to account for the unexpectedly low abundances of many mRNAs. Obviously, something was missing from the published model. Our mRNA decay rates (Fig. 7A, step 6) already included both intra-nuclear and cytoplasmic degradation of the mature mRNA, so differences in nuclear export of mRNA could not explain the discrepancies between the model and reality. We identified transcription, polyadenylation and precursor decay as possible variables.

**Table 3:** The reactions in the model and their rate equations

Rate	For reaction	Rate equation or value
v_1_	Transcription into precursor	0.24 min^−1^
v_2_	5′ *trans*-splicing of unspliced precursor	*k*_2_ × [unsplicedprecursor]
v_3_	3′ poly-adenylation	*k*_3_ × [5′splicedprecursor]
v_4_	Degradation of unspliced precursor	(*k*_4_ + *μ*) × [unsplicedprecursor]
v_5_	Degradation of 5′ *trans*-spliced precursor	(*k*_5_ + *μ*) × [5′splicedprecursor]
v_6_	Degradation of mature mRNA	(*k*_6_ + *μ*) × [mRNA]

### Modelling predicts an important role for co-transcriptional mRNA decay

The first issue that we needed to address was the assumption that the transcription rate is constant within, and between, transcription units. It has previously been shown that genes that encode mRNAs that persist after heat shock are located towards the end of transcription units (Kelly *et al.*, 2012). This presumably enables synthesis of the mRNAs to continue for some time after stress-induced shut-off of transcription initiation (Kelly *et al.*, 2012). The distance of a gene from the transcription start site also bore a relationship with cell cycle control (Kelly *et al.*, 2012). We therefore considered the possibility that the position of the gene within the transcription unit was having an effect on the mRNA abundance at steady state. To assess this, we plotted the half-life : abundance ratios for each gene across the chromosomes. The plot for chromosome 10 is in Supplementary Fig. S9. Although more detailed analysis might reveal some trend for particular mRNA classes, we found no gradient within transcription units, and no differences between transcription units. We therefore did not add variations in transcription rate to the model.

Next, we added a step that was obviously missing from the published model: polyadenylation (step 3 in Fig. 7A). This was assigned the same kinetics as for *trans* splicing (Ullu *et al.*, 1993). We assumed that it, like *trans* splicing, was competing with precursor degradation – in this case, degradation of a primary transcript that has been 5′ *trans* spliced, but not yet polyadenylated (step 5 in Fig. 7A).

Meanwhile, we discovered that the correlation between half-life and abundance was much better for shorter mRNAs than for longer ones (Fig. 6E and F and Fig. S10A), and that the shortest mRNAs fitted the published model relatively well (Fig. 6E). Longer mRNAs were on average less abundant than shorter ones with the same half-life (Supplementary Fig. S10B). We concluded that at least one of the steps in the model must include a negative influence of mRNA length.

We now set up a simulation that incorporated variations in processing, splicing and polyadenylation rates as well as in mRNA decay, and that included a negative influence of mRNA length. Splicing and polyadenylation were varied somewhat conservatively such that the half-time for each step was 2 ± 1.5 min. To mimic length-dependent co-transcriptional destruction we multiplied kinetic constant *k*_5_ (degradation of the *trans* spliced precursor) by a factor of (mRNA length in nt)/600. (The number 600 was chosen empirically.) Our rationale for this was that a longer transcript has more chance to be degraded before it is processed, since it takes longer for the polymerase to make a precursor that contains the downstream *trans* splicing signal. In addition, slow (inefficient) polyadenylation of an mRNA should also increase the likelihood of co-transcriptional destruction.

We now used this extended model to simulate steady-state abundances for transcripts with varying lengths, half-lives, 5′ *trans* splicing and 3′ polyadenylation kinetics, using the known length distribution for the mRNAs and a half-life distribution based on our measurements. The results are shown in Fig. 7B. We then compared the simulated data for each length group with the real data (Fig. 7C–E). There was a good qualitative agreement between simulations and the data. In the simulations the goodness-of-fit (R^2^) for the relation between mRNA half-life and abundance decreased with increasing transcript length, and in both the simulations and in the experimental data, abundance was highest for the short transcripts and lower for the longer ones. The real data, though, had bigger variation (lower R or R^2^) than the simulations. This may hint at additional regulation, or a need to adjust the parameters further. For instance, compared with the extended model, in the real dataset the intermediate length group (Fig. 7D) had more transcripts that were stable (half-life > 16′) and showed very low abundance. These might be mRNAs that are polyadenylated slower than the maximum half-time of 3.5 min that we allowed for in the model. (Unfortunately we could not model using the real distribution of processing times because these could not be measured using the RNASeq approach.) The model also predicted a population of long, very unstable transcripts with lower abundance than was seen in the data (less than one mRNA in every two parasites) (Fig. 7E). These would be very unstable, long transcripts that are processed slowly. It is possible that this particular combination of properties (long and unstable) is rare.

We also tested the effect of a theoretical length-dependent 3′ polyadenylation step – although it is difficult to imagine a mechanism for this (*k*_3_ decreasing with increasing transcript length). This also resulted in the linear relationship between mRNA half-life and abundance being poorer for longer transcripts but the effect was not as pronounced as seen when we allowed for co-transcriptional degradation (Supplementary Fig. S11A). Notably, if we also removed degradation of the 5′ *trans* spliced precursor (step 5), we obtained a virtually perfect relation between half-life and abundance for all lengths (Supplementary Fig. S11B). This is a consequence of the steady-state assumption. Simulations showed that without degradation of the 5′ *trans* spliced precursor, if transcription were induced from an ‘off’ state, slower 3′ polyadenylation lowered the mature mRNA abundance in the initial phase, but did not affect the abundance when the steady state was reached (not shown). If degradation of the un-spliced precursor was removed as well, a perfect correlation between abundance and half-life was obtained (Supplementary Fig. S11C). The modelling thus yielded the important conclusion that length-dependent variations in processing can only affect steady state mRNA abundance if processing is competing with degradation of the partly processed intermediates.

## Discussion

At the start of this study it was already known that the rate of mRNA decay is an important determinant of trypanosome mRNA abundance, but detailed quantitative information, including time-courses for the degradation, was available only for a very small number of mRNAs (Table 2). We have now provided transcriptome-wide decay kinetics for both bloodstream and procyclic forms, and have developed a detailed model for gene expression. We could confirm the important role of mRNA decay in determining steady state mRNA levels. We also found that a previously unrecognized step in processing has to be present to explain the steady-state mRNA abundances we measured. This additional step is most likely co-transcriptional degradation. Differences in mRNA maturation rates can only affect mRNA abundance if co-transcriptional degradation is also occurring.

The kinetics of mRNA decay fell into at least three different patterns. The ‘fast-slow’ group of mRNAs showed initial fast degradation, followed by a slower phase. This is also seen, in bloodstream forms, for mRNAs bearing the 3′-UTR of the mRNA encoding the major surface protein EP procyclin; in that case, the rapid degradation phase occurs without deadenylation and is dependent on the 5′–3′ exoribonuclease XRNA (Li *et al.*, 2006; Schwede *et al.*, 2009). Those *EP* mRNAs that are not attacked in this way are deadenylated more slowly, then degraded by XRNA and the exosome (Irmer and Clayton, 2001; Haile *et al.*, 2003; Li *et al.*, 2006; Schwede *et al.*, 2009). Depletion of the exosome caused a slight delay in the initiation of *EP* mRNA degradation (Haile *et al.*, 2003), suggesting some cross-talk between the 5′ and 3′ pathways. To see if this pattern was general we examined previous RNASeq results for parasites defective in different steps of mRNA decay. The fast-to-slow method of decay was indeed the predominant one in the case of mRNAs whose half-lives were increased after XRNA depletion (Manful *et al.*, 2011) (Fisher's test *P*-value << 0.01) (Supplementary Table S2, sheet 2), suggesting that the fast-to-slow pattern is diagnostic for XRNA-dependent, deadenylation-independent decay. However, this mode was also predominant for mRNAs whose decay had been significantly affected by exosome (RRP45) depletion (Fadda *et al.*, 2013) (Fisher's test *P*-value << 0.01) (Supplementary Table S2, sheet 2).

The ‘slow-fast’ group of mRNAs showed degradation rates that increased with time. This pattern has previously been observed by Northern blotting for numerous long-lived kinetoplastid mRNAs (for some references see Table 2). We speculated that ‘slow-fast’ kinetics could be understood in terms of stochastic (non-processive) deadenylation. Indeed, alpha and beta tubulin and histone H4 have slow-fast kinetics in bloodstream forms, and show decreases in overall length prior to mRNA decay (Schwede *et al.*, 2008). Degradation of nearly all mRNAs is to some extent affected by depletion of the CAF1/NOT complex (Fadda *et al.*, 2013), so we could not use RNASeq data from such cells to look for differential effects. However, transcripts affected by depletion of the deadenylase PAN2 (Fadda *et al.*, 2013), were predominantly degraded in the slow-fast method (Fisher's test *P*-value << 0.01) (Supplementary Table S2, sheet 2). This supports the idea that the slow-fast pathway reflects deadenylation prior to decay. As expected, abundant mRNAs always have relatively long half-lives; we could now show that they also often decay with slow-fast or exponential kinetics.

We found 179 mRNAs in which mRNA decay played a dominant role in developmental regulation. Most likely, the number is higher, since 200 regulated mRNAs showed such a low abundance in one life cycle stage that the half-life could not be measured. The mRNAs can also be subdivided into classes according to whether they are preferentially affected by RNAi targeting the 5′ or 3′ degradation in bloodstream forms, or according to their degradation pattern (fast/slow, slow/fast or exponential) in both forms. Combined with our previous analyses of the effects of inhibiting particular degradation pathways, we now have clear groups of mRNAs that are subject to similar degradation control, and which might therefore share sequences that govern that control. For example, half of the RNAs that showed lower abundances and half-lives in bloodstream forms than in procyclics (Supplementary Table S3, sheet 7) switched from the fast-slow to the slow-fast or exponential modes of degradation. We suggest that in bloodstream forms, some of these mRNAs are bound by proteins that recruit the decapping enzyme or XRNA; while in procyclic forms, the mRNAs are bound by different proteins and degraded by the slower, deadenylation-dependent route. Previous attempts to computationally predict regulatory sequences were limited by the resolution of the microarray data used and the fact that it showed only steady-state mRNA levels (Mao *et al.*, 2009; Queiroz *et al.*, 2009; Najafabadi *et al.*, 2013). The higher resolution of our new data may now facilitate identification of regulatory motifs.

Our experimental results showed that degradation of the mature mRNA is not the only factor that contributes to steady state abundance. Most notably, for a given half-life range, mRNA abundances decreased with length. There is clear evidence that mRNA processing is important in trypanosome gene expression regulation (Michaeli, 2011): experiments with reporter genes have shown strong variations in splicing efficiencies depending on the sequences preceding the AG dinucleotide (Kapotas and Bellofatto, 1993; Avliyakulov *et al.*, 2003; Siegel *et al.*, 2005), and when alternative splice sites are available, different ones may predominate in procyclic and bloodstream forms (Nilsson *et al.*, 2010; Siegel *et al.*, 2010; Zhang *et al.*, 2010; Rettig *et al.*, 2012). Slow polyadenylation may also explain previous reports of kinetoplastid mRNA regulation by sequences in downstream intergenic regions (Ramamoorthy *et al.*, 1995; Brooks *et al.*, 2001; Fluck *et al.*, 2003). Our results now show that under steady-state conditions, the processing efficiency can only influence mRNA abundance if processing is competing with precursor degradation. Moreover, the length dependency of mRNA abundance can be explained by competition between length-dependent mRNA precursor degradation and polyadenylation.

Our kinetic model does not yet account for all of the features of the data. There is a variety of possible reasons for this. First, we restricted ourselves mainly to varying known kinetic parameters within the limits that are suggested by the available measurements. The simulation results might be improved if we allowed for more variation in these parameters, or added additional variables for which there is currently no experimental evidence. For example, polyadenylation was assigned the same rate as *trans* splicing, but may in fact be slightly slower, since it is thought to occur after splicing has been completed (Ullu *et al.*, 1993). Second, in considering the effects of transcript length on degradation of the spliced precursor, we have opted for a simple length-dependence, which largely leaves transcripts < 600 nt unaffected by intermediate degradation. We could have changed the boundary chosen, or made this dependence more complex. Third, the model was evaluated at steady state, but we cannot rule out that pre-steady-state kinetics influenced the experimental results. If there are variations in the overall transcription initiation rate during the cell cycle, some slowly processed mRNAs may never reach steady state.

The detailed mechanism of trypanosome co-transcriptional degradation is currently unknown. Co-transcriptional degradation certainly occurs in other systems, although the details may not apply in trypanosomes. In Opisthokonts, the nuclear quality control machinery is triggered by failure of mRNA capping, and by failure to splice or polyadenylate a terminated mRNA (Schmid and Jensen, 2010; 2013; Kilchert and Vasiljeva, 2013). 5′->3′ degradation by Xrn2/Rat1 requires a free 5′ end, and has mainly been studied in relation to transcription termination beyond polyadenylation sites (Schmid and Jensen, 2010; 2013; Kilchert and Vasiljeva, 2013). With trypanosome polycistronic transcription, most mRNAs have no free 5′ end, since the mature 5′-end is created by trans-esterification, and termination is relevant only for a tiny proportion of genes. Polyadenylation might, however, fail after downstream *trans* splicing has been completed, and the resulting free 3′-ends should be attacked by the nuclear exosome (Cristodero and Clayton, 2007; Etheridge *et al.*, 2009). There is, though, no reason to think that this would be more efficient for long mRNAs than for short ones and in fact, depletion of the exosome actually causes preferential *loss* of longer mRNAs (Fadda *et al.*, 2013; Jha *et al.*, 2014). The NOT deadenylation complex is associated with RNA polymerase II in mammals, but it is thought to promote (not inhibit) elongation (Reese, 2013). Previous experiments that aimed at characterization of RNA polymerase II subunits and transcription factors (e.g. Lee *et al.*, 2009) were not designed for detection of proteins present in sub-stoichiometric amounts, but after tandem affinity purification of the NOT complex, we detected two peptides from a putative polymerase II elongation factor (Tb927.2.3580) (Färber *et al.*, 2013).

Our data are best explained by the existence of an endonuclease that intermittently cleaves the associated mRNA precursor. This is highly speculative since there is, so far as we know, no real precedent for such a degradation process (Schmid and Jensen, 2010; 2013; Kilchert and Vasiljeva, 2013). To recognize the precursor specifically, we would expect the endonuclease to be associated either with chromatin, or with the elongating RNA polymerase II complex. One consequence of such a mechanism would be that a decrease in the polymerase elongation rate would increase the time that the precursor is accessible for digestion. Indeed, after depletion of trypanosome CTR9, a probable transcription elongation factor (Ouna *et al.*, 2012), the abundances of long mRNAs were drastically decreased (Fadda *et al.*, 2013; Jha *et al.*, 2014). In animal cells and yeast, elongating RNA polymerase II pauses, and even backtracks (Darzacq *et al.*, 2007; Churchman and Weissman, 2011). We speculate that such pauses might, in trypanosomes, be a trigger for cleavage without detachment of the polymerase, and could even be influenced by the DNA sequence. This would provide an additional means to regulate the formation of mRNA in the absence of any control of transcription initiation.

The simplicity of the trypanosome system has made it possible to construct a mathematical model of mRNA control which has revealed novel aspects and refined our view of the likely contribution of mRNA processing and degradation to mRNA abundance. When combined with ribosome profiling to determine translation efficiency, our measurements will enable us to mathematically model trypanosome gene expression from DNA to protein.

## Experimental procedures

### Sample preparation

Bloodstream and procyclic form trypanosomes of the Lister 427 strain, expressing the tet repressor, were used; the bloodstream forms were the 2T1 line described in (Alsford *et al.*, 2005), while procyclic cells expressed the tet repressor from plasmid pHD2060. Bloodstream forms were cultured in HMI-9 medium containing 20% FCS, to a density of 1.0–1.2 × 10^6^ cells ml^−1^; procyclic forms were cultured in MEM-Pros to a density of 2 × 10^6^ (experiment 1) or 1.3 × 10^6^ (experiment 2) cells ml^−1^. For the pre-mRNA decay assay, transcription was inhibited briefly by incubating the cells with Actinomycin D (10 μg ml^−1^) and samples were harvested immediately (equivalent to a 5 min incubation); the bloodstream form experiment was done twice; for procyclics a single experiment was done. For the mRNA decay assay, cap methylation was inhibited by adding 1 μg ml^−1^ Sinefungin for 5 min to prevent splicing (t0 samples), then Actinomycin D was added for up to 120 min for bloodstream forms and 240 min for procyclics. All samples are listed in Supplementary Table S1. The parasites were harvested by centrifugation for 5 min (all times given include the centrifugation time); RNA was extracted using peqGOLD-Trifast (peqLab) and depleted for rRNA using Ribominus Eukaryotic kit for RNA-seq (Invitrogen, no. 10837-02 or -08). The RNA was then sequenced (Library preparation at the BioQuant facility, Heidelberg and HiSeq at EMBL). The first set of bloodstream-form decay samples was measured using Illumina GAIIX and all remaining samples with Illumina HiSeq (Supplementary Table S1). All data are available at the NCBI Sequence read archive, with accession number SRP042959, and a simplified version of the results has been sent to TritrypDB. We cannot rule out the possibility that the Sinefungin or Actinomycin D treatments influenced the mRNA decay rates, especially after long incubation times, but at present alternative methods are not available.

### RNA decay analysis by Northern blotting

To determine the overall mRNA decay rate, aliquots of the sequenced samples were separated by agarose gel electrophoresis, blotted and probed with a spliced leader probe, using the rRNA signal for normalization, as previously described (Fadda *et al.*, 2013). The measured signal was used to fit an exponential decay curve with R (Supplementary Fig. S4) and the values corresponding to the curve were used to normalize the RNAseq decay data.

### Data processing

Reads were aligned to the *T. brucei* 927 reference genome, and read counts were extracted using Bowtie, SamTools and custom-made PERL scripts as previously described (Fadda *et al.*, 2013). Read counts per ORF were first normalized for the total number of counts (RPM, reads per million reads). In order to build the libraries, the same amount of input RNA was used for each sample; and similar numbers of read counts were obtained for each library. After addition of transcription inhibitors, the proportions of the unstable mRNAs go down (giving a decrease in RPM) while the proportions of the stable RNAs go up (giving a decrease in RPM). The stable mRNAs seem to increase because the overall reduction in mRNA levels is not reflected in the RNASeq data. This necessitates the use of an external control to detect – and correct for – the decline in mRNA quantities in the cell with time. We could not normalize using endogenous stable RNAs, because they were excluded during the ribo-minus and size selection steps of library preparation. To measure global mRNA decay we therefore used Northern blots to assay the decline of the spliced leader (*SL*) signal, as previously described (Fadda *et al.*, 2013). Then, to account for the decreasing total amounts of RNA during the decay assay, the RPM for each time point were multiplied by the corresponding SL signal at that time. ORFs with an average of less than 50 reads at time 0 were excluded from the half-life analysis. Lastly, the read counts were calculated as a ratio to time point 0 (t0). This format was used as input for a C++ code to calculate half-lives. Using the equations described by Deneke *et al.*, (2013) we allowed for three alternative decay patterns: simple exponential, initially fast, then slower (fast-slow, equation 9 of Deneke *et al.*, 2013) and initially slow, then fast (slow-fast, equation 10 of Deneke *et al.*, 2013). For the calculations each measurement was treated as an independent data point. Differential expression was analysed by DESeq (Anders and Huber, 2010).

To measure splicing kinetics, read counts were extracted for the 20mer upstream of all known splice acceptor sites for each gene ( Kolev *et al.*, 2010; Nilsson *et al.*, 2010; Siegel *et al.*, 2010; Zhang *et al.*, 2010 and our data), as well as for each coding region. If the read counts for the 20mer were equal to, or more than, the length-normalized read counts in coding region, we assumed that the splice site was not being used in our sample, and the data for the site were removed; we also removed sites if no reads existed in the 20mer region in the control (untreated) sample. Finally, zero values in the 20mer region in Actinomycin D samples were converted to 1 read. Read counts per 20mer were normalized to the read counts per coding region of the corresponding gene in each sample. The ratio of the normalized 20mer counts in the treated/untreated samples was used to calculate a pre-mRNA half-life based on an exponential model. While interpreting the results, we attribute this to splicing, although strictly speaking pre-mRNA degradation also contributes. Probability density plots were done using a kernel density function contained in the R package ‘ggplot’.

To calculate gene copy numbers, we first did a control for uniformity of coverage. We mapped the reads from the reference genome against itself using reads available from Dr A. Ivens (University of Edinburgh). Allowing each read to map only once should yield a bell-shaped coverage plot. Next we allowed the reads to map up to 300 times and calculated RPKM for each gene. We assumed that the modal RPKM value (M) corresponded to a gene copy number of 1, since most genes should be unique. Next, we aligned the reads from sequenced, fragmented DNA from our lab strain, and mapped them to the reference genome, allowing each read to align only once. We again calculated the modal value (M′). If the number of reads from a gene was M′, then that gene has the same copy number as in the reference; if M′ has a value *n* × M, then the strain has *n* times the number of copies in the reference.

### Calibrating decay models

The code for decay calibration is written in C++ and is provided as Supplementary file S1. We used the equations described by Deneke *et al.*, (2013) to model yeast mRNA decay; that paper explains the mathematical details. Complex, non-exponential mRNA degradation is modelled by a Markov chain with 5 states; each state represents a different biochemical process of mRNA degradation, where the molecule is likely to be degraded at any state. The rate of transition between states is assumed to be the same for all states and is denoted as *λ*. The degradation rate from any state is denoted as *μ*. In one variant of the complex model the initial rate of degradation *μ*_1_ is assumed bigger than all subsequent rates, denoted *ν*. This is described by the parametric density Equation 9 (Deneke *et al.*, 2013) and referred to here as ‘fast-slow’ model. The other model variant assumes all degradation rates except the last to be equal, and the last rate *μ_n_* ≡ *ν* > 0. This is described by the parametric density Equation 10 (Deneke *et al.*, 2013) and here referred to as ‘slow-fast’ model. We calibrated and compared three models: the exponential model, ‘fast-slow’ and ‘slow-fast’. The calibration procedure worked in several stages. In the first stage we calibrated each model. The fitting procedure using non-linear least squares which we used (Dennis *et al.*, 1981) is extremely fast but suffers numerically from multiple local minima. These local minima are obtained from different ‘initial parameter guesses’. For the three-parameter models we chose to calibrate the model using 125 different starting points, lying on a mesh between 0 and 3.0 with 5 points per axis. For each calibration we stored the residual model error and the calibrated parameters. In the next stage we ran a rejection/acceptance procedure for each of the parametric forms, and for each of a form's 125 models. The rejection criteria were subjective, but were designed to reject models that were completely implausible, or had collapsed to simpler models. The rejection criteria for each model are found in the function *includeTentativeSolution()*. As an example, for a calibrated model of Equation 9's density we have parameters *λ*, *μ*, *ν* (Deneke *et al.*, 2013). The model is rejected if *λ* or *ν* are 0, or if any of the parameters have become infinite. The next stage of calibration chooses the best fitting accepted model for each density type. We call the three best-fit models the three ‘candidate’ models. The last stage of calibration selects the best-fit model from among the candidate models. The null hypothesis is that the distribution is Exponential E. For a candidate model *C* ≠ *E* we look at the relative
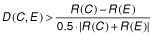
where *R*(*M*) is the residual sum of squares error from fitting with model *M*. If *D*(*C*;*E*) > 5% we rejected the null hypothesis and accepted *C*. The choice of threshold is essentially arbitrary. Deneke *et al.* (2013) used a boundary of 10%, we chose 5% after visual examination of individual examples. Using the lower threshold leads to a lower proportion of curves being designated as exponential. We did not favour either the Equation 10 or Equation 9 density over each other and simply took the candidate from the two with the minimal residual. The ‘half lives’ for the mRNAs with non-exponential decay in all the Tables are the time at which half of the mRNA is left (the value of time *T* where 50% of the distribution lies above *T*, and 50% lies below *T*).

### Modelling mRNA processing and decay

The *PGK* mRNA model developed in Haanstra *et al.* (2008) was initially used. Then we made a new, modified version which applies to any transcript. The new model was extended to include polyadenylation and length-dependent degradation of the *trans* spliced intermediate. Model simulations were done, and extensions were made, in the open source software COPASI v.4.9.45 (Hoops *et al.*, 2006). Figure 7A shows a graphical representation of the reactions in the model, and their rate equations are listed in Table 1.

The constants *k* in the equations relate to half-lives (HL) of the corresponding species via
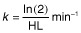


For *k*_4_, *k*_5_ and *k*_6_ this relation has been explicitly included in the model, which enables us to affect the *k's* by changing the half-life through random sampling (see below).

*k*_2_ and *k*_3_ each have a default value of 0.41 (half-life of 1.7′) (as in Haanstra *et al.*, 2008). We assumed *k*_4_ and *k*_5_ to be equal to each other and took a value of 0.08 min^−1^ which is based on the previously measured half-life of the *PGK* precursor after inhibition of both transcription and splicing by Actinomycin D and Sinefungin (Haanstra *et al.*, 2008). μ is the specific growth rate and is 0.0019 min^−1^ (a population doubling time of 6 h).

The differential equations that describe the changes in species concentrations over time in the model are:
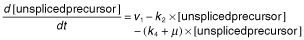

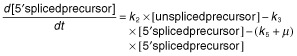

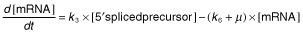


Concentrations are in molecules cell^−1^.

To simulate effects that depend on the length of the mature mRNA, the relevant constant was multiplied or divided by (length in nt)/(600 nt): For length-dependent degradation of the 5′ spliced intermediate, *k*_5_ was multiplied by length/600 so that longer transcripts would be affected faster. For length-dependent polyadenylation, *k*_3_ was multiplied by 600/length so that polyadenylation would be slowed down for longer transcripts.

The model was used to simulate steady state abundances for individual transcripts (i.e. with their own values for the kinetic constants). To this end, 5510 transcripts were simulated by

- Sampling the half-life of the un-spliced precursor in the 5′ *trans* splicing reaction from a normal distribution with a mean of 2′ and a standard deviation of 1.5′ (affects *k*_2_),
- Sampling the half-life of the 5′ spliced precursor in the 3′ poly-adenylation reaction from a normal distribution with a mean of 2′ and a standard deviation of 1.5′ (affects *k*_3_; only used when *k*_3_ was not multiplied by a length-dependent factor), and
- Sampling the length of a transcript from a uniform distribution for short (551 transcripts in the range 400–1000 nt), intermediate (4408 transcripts in the range 1000–3000 nt) and long (551 transcripts in the range 3000–20000 nt) transcripts

For each length range, the half-life of the mature mRNA (affecting *k*_6_) was sampled from a uniform distribution within the half-life range in each bin in Fig. 3, except that for the first bin, the half-lives ranged between 1–5′. The number of transcripts in the length range for each bin was taken proportionally to the distribution of lengths of the overall dataset (10% short, 80% intermediate and 10% long transcripts).

In some cases, the combination of parameters did not result in a steady state and the final number of solutions was therefore lower than the number of simulations.
