# Supplementary material for: Transcriptome-wide analysis of trypanosome mRNA decay reveals complex degradation kinetics and suggests a role for co-transcriptional degradation in determining mRNA levels
Source: Mol Microbiol. 2014 Sep 15;94(2):307–26. doi: 10.1111/mmi.12764 (PMC4285177; doi:10.1111/mmi.12764)
Supplement: Supplementary file 1 — Supporting Information [file mmi0094-0307-SD1.pdf]

Supplementary Files and Tables

Supplementary File S1

C++ code for fitting of decay rates.

Supplementary Table S1

Details of all samples.

Supplementary Table S2

Simplified summary of the results.

Sheet 1 is a summary of the results from this paper. BS: bloodstream form; PC: procyclic form. HL: half-life. Gene classes were determined from TritrypDB and by manual annotation. Note that in this Table, only unique genes from the list in Siegel et al (Siegel *et al.*, 2010) are included. mRNA lengths are from TritrypDB; for filtered lengths, mRNAs without annotated processing sites have been removed. Manful gene copy no is from (Manful *et al.*, 2011); 2T1 Gene copy no: results for the strain used in this paper, obtained from random shotgun reads of genomic DNA. RNAs/cell: number of RNAs per cell. Shortest pre-mRNA HL: half-life obtained for reads at the possible splice sites, shortest one chosen.

In Sheet 2 the results for half-life are compared with those from Manful et al. (Manful *et al.*, 2011), and results for mRNA/cell with Vasquez et al. (Vasquez *et al.*, 2014). We also include our own results for poly(A)+ mRNA abundances from bloodstream-form and procyclic-form trypanosomes (average of two measurements) (Singh *et al.*, 2014), and the effects on half-life of depleting XRNA (Manful *et al.*, 2011) or PAN2 or RRP45 (Fadda *et al.*, 2013).

Supplementary Table S3

Developmental regulation and half-life

Sheet 1: Raw data (read counts) for samples that had not been treated with Actinomycin D. For bloodstream forms these are 2 each for untreated and 5min Sinefungin; for procyclics, there were 1 untreated and 3 treated with Sinefungin (Supplementary Table S1). The Sinefungin samples could be included because the variability between the two groups of life stage samples was much larger than internal variability, as revealed by principal component analysis (not shown).

Sheet 2: Statistical analysis of developmental regulation for total (non poly(A)-selected) mRNAs. P-values and p-adjusted (corrected for multiple testing) values were calculated according to DESeq (see methods). The results from previous analyses are also included (see text). Bloodstream-specific values (>2x) are in red and procyclic-specific (>2x) in blue. The baseMean is the number of reads divided by the size factor (normalization constant) of the sample, as calculated using DESeq.

Sheet 3: Significantly developmentally regulated genes in our analysis compared with results from the analyses of Siegel *et al.* (Siegel *et al.*, 2010), Jensen *et al.* (Jensen *et al.*, 2009) and Vasquez *et al.* (Vasquez *et al.*, 2014). For colour code and further details see text box.

Sheet 4: Genes that were at least 1.5-fold regulated (in a similar fashion) in at least 3 of the 4 available RNASeq datasets. The colour code is the same as in Sheet 3. "No regulated" indicates the number of experiments in which at least 1.5-fold regulation (in the same direction) was seen.

Sheet 5: Genes that were removed in order to plot Figure 5A and B, because, as outliers, they unduly influenced the results. They were either > 5 fold different between the poly(A)+ and ribo-datasets (upper set) or > 5 fold different between the Siegel poly(A)+ and Fadda datasets (lower set).

Sheet 6: Half-lives related to mRNA abundances for all unique open reading frames.

Sheet 7: mRNAs in which changes in mRNA decay clearly contribute to changes in abundance. These are mRNAs in which both the half-life and the abundance differ at least 2-fold between stages, in the same direction.

Sheet 8: mRNAs that show developmental regulation in abundance without corresponding changes in mRNA decay.

Sheet 9: Realistic half-life measurement (at least 2 min) available for only one life-cycle stage.

Supplementary Table S4

Raw data for mRNA degradation, half-lives and fit parameters for all genes

Sheet 1: Raw and normalised RPM (reads per million) data for procyclic forms. The first set of columns show the raw data. The second set in yellow are normalised RPMs. To obtain these the RPMs were first calculated based on the non-redundant set of genes, then normalized to the SL signal as explained in the text (columns labelled "SL norm"). After normalization to time zero (not shown) the reads were used as input to the C++ code. To plot the decay curve for a gene of interest, use the data from columns R - AG. The time point after Actinomycin D addition is indicated by the number after "t", for example t30 means 30 min.

Sheet 2: Raw and normalised RPM (reads per million) data for bloodstream forms. Details as for sheet 1.

Sheet 3: Half-life and fit parameters for all genes, procyclic forms. Eq9 = fast-slow decay; Eq10 = slow-fast decay; lambda = rate of transition between states; mu = rate of degradation; nu = rate of initial degradation; U = transcript lifetime; Error = least squares. See (Deneke *et al.*, 2013) for details. Values for isoform-specific regions of the PGK (Tb927.1.700-720) and THT (Tb927.10.8440, 8530) genes are also included but the errors are very large, probably because the regions are quite short or include rather repetitive parts of the 3'-UTR.

Sheet 4: Half-life and fit parameters for all genes, bloodstream forms. Details as in Sheet 3.

Supplementary Table S5

Functional group half-lives and developmental regulation

A Student t-test was performed for each functional class between the two life stages. Significant differences (p-value < 0.05) are in blue, and the extent of difference (bloodstream / procyclic ratio of mean half-life (HL) values) is coded in shades of green.

Supplementary Figure legends

Supplementary Figure S1

Developmental regulation results from different analyses

The regulation is plotted on a $\log_2$ scale (0= no regulation, 2 = 4x regulation, -2 - 1/4, etc.) Correlation coefficients and equations are also for $\log_2$-transformed data and were plotted using Microsoft Excel. Each spot represents an individual unique open reading frame. All outliers were

included. Datasets were: Fadda: this paper, rRNA-depleted RNA, sheared and 300nt-size-selected before cDNA synthesis; Singh (Singh *et al.*, 2014) poly(A)+ RNA, sheared and 300nt-size-selected before cDNA synthesis; Siegel (Siegel *et al.*, 2010) poly(A)+ RNA, not sheared before cDNA synthesis; Vasquez (Vasquez *et al.*, 2014) poly(A)+ RNA sheared and 20nt-size-selected before cDNA synthesis. The magenta dashed line is perfect correlation.

In theory, differences due to methods of RNA and library preparation, and sequencing, should cancel out when ratios are calculated. The fact that the correlation in (A) is much better than cross-laboratory correlations suggests that culture conditions and the precise parasites used are important.

A) Singh poly(A)+ vs. Fadda, rRNA-depleted

B) Singh poly(A)+ vs. Siegel poly(A)+

C) Fadda rRNA-depleted vs. Vasquez poly(A)+

D) Fadda, rRNA-depleted vs. Siegel poly(A)+

E) Singh poly(A)+ vs. Vasquez poly(A)+

F) Siegel poly(A)+ vs. Vasquez poly(A)+

Supplementary Figure S2

Comparisons of absolute RNA abundances between datasets

The numbers of mRNA per cell are plotted on a $\log_2$ scale but the scale has been converted to absolute numbers for ease of viewing. Correlation coefficients and equations are for the $\log_2$-transformed data; coefficients for non-log-transformed data are in parentheses. Results are from: Fadda - this paper and Manful (Manful *et al.*, 2011) either poly(A)+ or rRNA-depleted RNA, sheared and 300nt-size-selected before cDNA synthesis; Singh (Singh *et al.*, 2014) poly(A)+ RNA, sheared and 300nt-size-selected before cDNA synthesis; and Vasquez (Vasquez *et al.*, 2014) poly(A)+ RNA sheared and 20nt-size-selected before cDNA synthesis. The within-lab correlations are best, especially those performed most recently (Singh vs Fadda). The Manful dataset was generated using older library construction and sequencing technology. The magenta dashed line is perfect correlation.

A) Bloodstream forms, Manful vs. Fadda, rRNA-depleted RNA.

B) Bloodstream forms, Singh poly(A)+ vs. Manful poly(A)+

C) Procyclic forms, Singh poly(A)+ vs. Fadda rRNA-depleted

D) Bloodstream forms, Singh poly(A)+ vs. Vasquez poly(A)+

E) Procyclic forms, Fadda rRNA-depleted vs. Vasquez poly(A)+

F) Bloodstream forms, Fadda rRNA-depleted vs. Vasquez poly(A)+

Supplementary Figure S3

Half-lives of pre-mRNAs

Precursor half-lives were placed into categories as indicated, and the number of mRNAs in each category was counted. For mRNAs with more than one annotated splice site, results are shown only for the site with the shortest half-life.

Supplementary Figure S4

SL signal calibration

Overall mRNA decay was determined by fitting an exponential curve into data points of 3 biological replicates (and 2 technical replicates for BF) of the Northern SL hybridization blots. The values used to normalize the RNAseq data corresponding to the curve are: bloodstream forms t0 0.92, t5 0.82, t10 0.73, t20 0.59, t30 0.46, t60 0.23, t120 0.06. procyclic forms t0 0.92, t15 0.79, t30 0.68, t60 0.50, t120 0.27, t180 0.15, t240 0.08.

Supplementary Figure S5

Curve-fitting for selected genes

Individual relative mRNA levels are shown for the different time points. Different curves were fitted as indicated. In each case, the exponential pattern was rejected in favour of a slow-fast or fast-slow model. Results are for:

A) Tb927.5.4170

B) Tb927.11.16310

C) Tb927.1.2330

D) Tb927.11.16600

E) Tb927.9.8850

F) Tb927.8.2810

Note that the data for E and F are also shown In Figure 2; we show them here as well because it is easier to compare the two curves. without the Northern and qPCR results,

Supplementary Figure S6

Comparison of the current half-life results with previous ones.

A. The half-lives obtained for bloodstream-form mRNAs in this paper are compared with those calculated in Manful et al., (Manful *et al.*, 2011), from incubation with Actinomycin D for 30-min. Stable mRNAs (half-life >120 min) were arbitrarily assigned a half-life of 240 min. Results are shown on a linear scale. The P value from a Students t-test (comparing the two datasets and counting only half-lives between 2 min and 120 min) was 1.4 E-37.

B. Same results as in (A), but all data are $\log_2$ transformed and the formulae and correlation coefficients are for the log-transformed data. The mRNAs are divided into three equally-sized groups according to half-life. The correlation is best for more long-lived mRNAs. This may be partly because the half-lives are nearer to the 30-min time point used in the Manful study. The very short-lived mRNAs yield very few read counts at the 30-min time-point, which makes half-life estimates based on that point alone inaccurate: note that no half-lives lower than 7 min could be measured.

Supplementary Figure S7

Comparison of RNAseq degradation curves with published Northern blot or qRT-PCR measurements.

RNASeq results are in black, Northern results in green and real-time qRT-PCR results are in cyan. The Northern and real-time qRT-PCR PCR data are those that were used to calculate half-lives listed in Table 2. The different symbols represent different biological replicates for qRT-PCR. For

Northerns, each symbol represents the average of at least 3 independent biological replicates, and the different symbols are from different replicate sets. For consistency, clear outliers in both datasets have been included. All results are for bloodstream forms.

Supplementary Figure S8

Regulation of abundance correlates poorly with regulation of half-life.

The steady-state mRNA abundance in bloodstream forms, divided by that in procyclic forms, is on the x-axis, while the corresponding half-life ratio is on the y-axis.

Supplementary Figure S9

The half-life - abundance ratio does not depend on chromosomal position

The Figure is a schematic depiction of chromosome 10. Genes were ordered according to their position on the chromosome. Transcription initiation (green and red bars) and termination points (blue bars) were assigned manually according to ORF direction and chromatin marks (Siegel *et al.*, 2009). The arrows show transcription direction and were also placed manually, and since some transcription units are short there may be some errors in the assignments. Purple bars: the half-life (in min) was divided by the number of mRNAs/cell/gene, and the result was $\log_2$ transformed. High bars therefore mean that the mRNA has unexpectedly low abundance for its half-life. A space means only that there is no data for this particular position, or that it was not in the unique gene set.

Supplementary Figure S10

The relationship between half-life and abundance

A. The mRNA lengths were extracted from TritrypDB and mRNAs lacking annotated splice or poly(A) sites were excluded. The coding sequences were then ranked according to the length and divided into 5 bins of equal size. Correlation coefficients were calculated, using log-transformed data, between the half-life and the number of mRNAs per cell per gene.

B. All mRNAs with half-lives between 8 and 16 minutes were extracted, ranked according to the length then divided into 5 bins of equal size (237 for procyclic forms, 435 for bloodstream forms). For each bin, the arithmetic mean of the abundance and the standard deviation are plotted. Note that given the sample size, standard errors would be too small to be visible on the plot. The mRNA abundances for each of bins 2-5 were significantly lower than those of bin 1 (Student T-test, P value < 0.02). Analyses using log-transformed data confirmed the relationship between length and abundance (not shown).

C. The number of mRNAs per cell per gene was plotted against the half-life for bloodstream forms, considering mRNAs that were less than 1kb long and had measured 5’ precursor half-lives of less than 3 min. The grey squares indicate the results of modelling steady-state mRNA abundance with the published model (Haanstra *et al.*, 2008) assuming a 5’-*trans* splicing half-time of 1 min. Note that the relatively high correlation coefficient for the data is partly caused by the fact that the sample size is smaller than in other plots.

D. The number of mRNAs per cell per gene was plotted against the half-life for procyclic forms, considering only mRNAs that were less than 1kb long and had measured 5’ precursor half-lives of less than 4 min.

Supplementary Figure S11

Effects of changing the model parameters

Results are displayed as for Figure 7, panels C-E. Transcript length, splicing half-life and half-life of the mature mRNA were sampled as for the results in Fig 7.

A. The constant for polyadenylation (k_3; 0.41 min^{-1}) was multiplied by (600nt)/(mRNA length in nt) and degradation of the 5'-trans spliced precursor (k_5) was kept constant at 0.08 min^{-1}.

B. The constant for polyadenylation (k_3; 0.41 min^{-1}) was multiplied by (600nt)/(mRNA length in nt) and degradation of the 5'-trans spliced precursor (reaction 5) was removed.

C. Similar to (B), but reaction 4 (degradation of the un-spliced precursor) was also removed

References

Deneke, C., R. Lipowsky & A. Valleriani, (2013) Complex degradation processes lead to non-exponential decay patterns and age-dependent decay rates of messenger RNA. *PLoS ONE* **8**: e55442.

Fadda, A., V. Färber, D. Droll & C. Clayton, (2013) The roles of 3'-exoribonucleases and the exosome in trypanosome mRNA degradation. *RNA* **19**: 937-947.

Haanstra, J., M. Stewart, V.-D. Luu, A. van Tuijl, H. Westerhoff, C. Clayton & B. Bakker, (2008) Control and regulation of gene expression: quantitative analysis of the expression of phosphoglycerate kinase in bloodstream form *Trypanosoma brucei*. *J. Biol. Chem.* **283**: 2495-2507.

Jensen, B., D. Sivam, C. Kifer, P. Myler & M. Parsons, (2009) Widespread variation in transcript abundance within and across developmental stages of *Trypanosoma brucei*. *BMC Genomics* **10**: 482.

Manful, T., A. Fadda & C. Clayton, (2011) The role of the 5'-3' exoribonuclease XRNA in transcriptome-wide mRNA degradation. *RNA* **17**: 2039-2047.

Siegel, T., D. Hekstra, L. Kemp, L. Figueiredo, J. Lowell, D. Fenyo, X. Wang, S. Dewell & G. Cross, (2009) Four histone variants mark the boundaries of polycistronic transcription units in *Trypanosoma brucei*. *Genes Dev.* **23**: 1063-1076.

Siegel, T., D. Hekstra, X. Wang, S. Dewell & G. Cross, (2010) Genome-wide analysis of mRNA abundance in two life-cycle stages of *Trypanosoma brucei* and identification of splicing and polyadenylation sites. *Nucleic Acids Res* **38**: 4946-4957.

Singh, A., I. Minia, D. Droll, A. Fadda, C. Clayton & E. Erben, (2014) Trypanosome MKT1 and the RNA-binding protein ZC3H11: interactions and potential roles in post-transcriptional regulatory networks. *Nucleic Acids Res* **in press**.

Vasquez, J.J., C.C. Hon, J.T. Vanselow, A. Schlosser & T.N. Siegel, (2014) Comparative ribosome profiling reveals extensive translational complexity in different Trypanosoma brucei life cycle stages. *Nucleic Acids Res* **42**: 3623-3637.

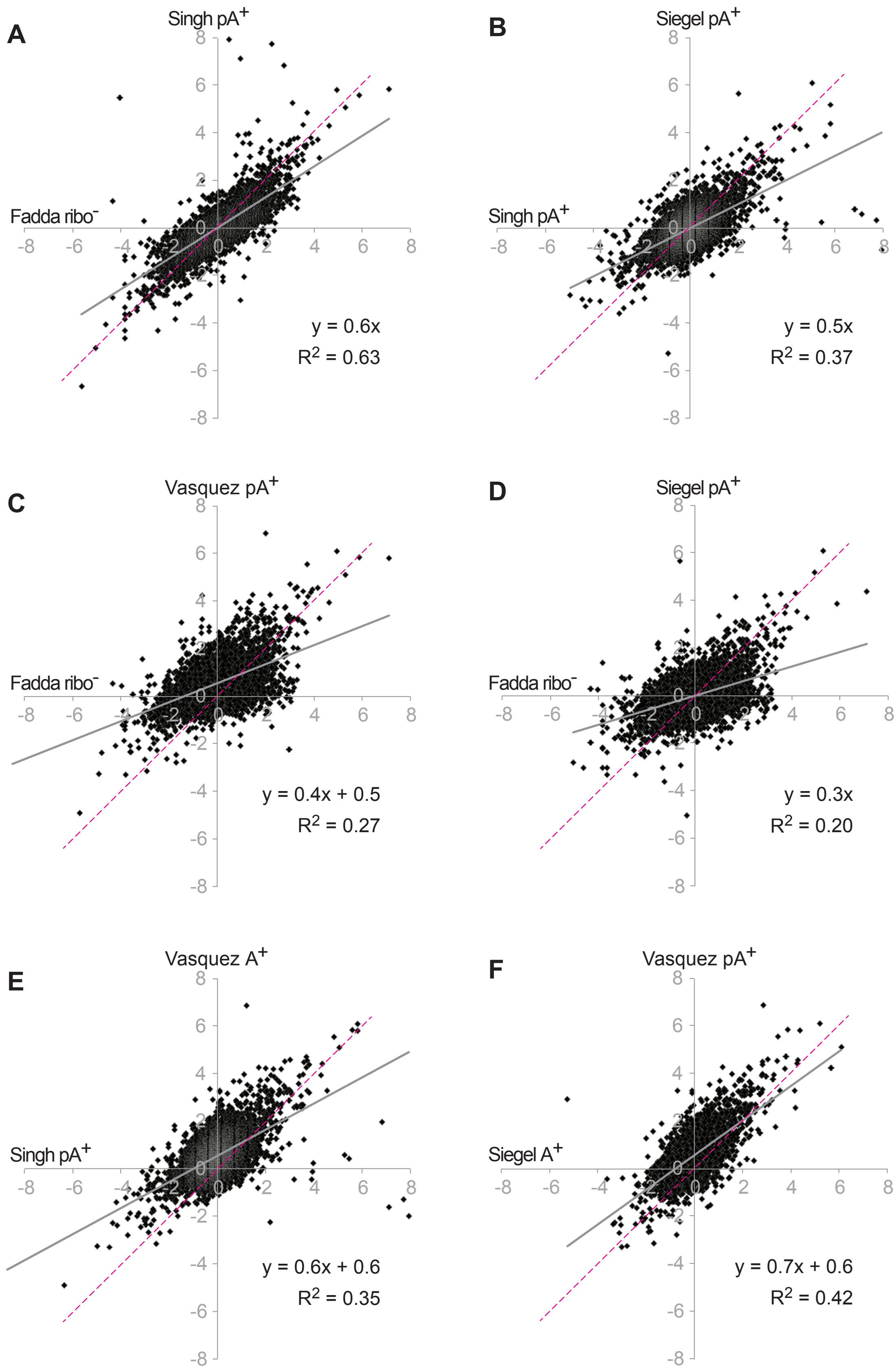

Supplementary Figure S1: Developmental regulation results from different analyses

The regulation is plotted on a log2 scale (0= no regulation, 2 = 4x regulation, -2 - 1/4, etc.) Correlation coefficients and equations are also for log2-transformed data and were plotted using Microsoft Excel. Each spot represents an individual unique open reading frame. All outliers were included. Datasets were: Fadda: this paper, rRNA-depleted RNA, sheared and 300nt-size-selected before cDNA synthesis; Singh (Singh et al., 2014) poly(A)+ RNA, sheared and 300nt-size-selected before cDNA synthesis; Siegel (Siegel et al., 2010) poly(A)+ RNA, not sheared before cDNA synthesis; Vasquez (Vasquez et al., 2014) poly(A)+ RNA sheared and 20nt-size-selected before cDNA synthesis. The magenta dashed line is perfect correlation. In theory, differences due to methods of RNA and library preparation, and sequencing, should cancel out when ratios are calculated. The fact that the correlation in (A) is much better than cross-laboratory correlations suggests that culture conditions and the precise parasites used are important.

A) Singh poly(A)+ vs. Fadda, rRNA-depleted
B) Singh poly(A)+ vs. Siegel poly(A)+
C) Fadda rRNA-depleted vs. Vasquez poly(A)+
D) Fadda, rRNA-depleted vs. Siegel poly(A)+
E) Singh poly(A)+ vs. Vasquez poly(A)+
F) Siegel poly(A)+ vs. Vasquez poly(A)+

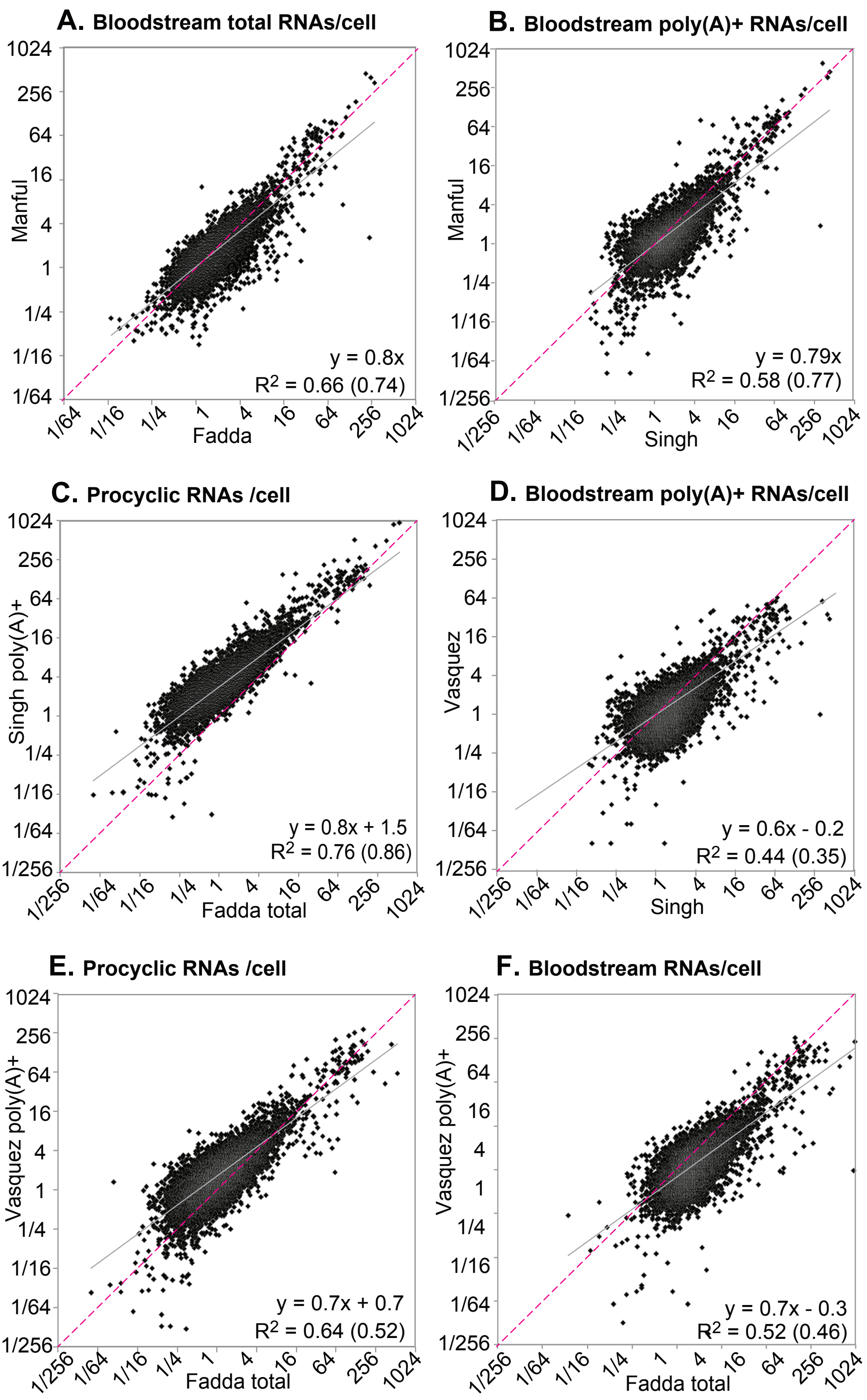

Supplementary Figure S2

Comparisons of absolute RNA abundances between datasets

The numbers of mRNA per cell are plotted on a log2 scale but the scale has been converted to absolute numbers for ease of viewing. Correlation coefficients and equations are for the log2-transformed data; coefficients for non-log-transformed data are in parentheses. Results are from: Fadda - this paper and Manful (Manful et al., 2011) either poly(A)+ or rRNA-depleted RNA, sheared and 300nt-size-selected before cDNA synthesis; Singh (Singh et al., 2014) poly(A)+ RNA, sheared and 300nt-size-selected before cDNA synthesis; and Vasquez (Vasquez et al., 2014) poly(A)+ RNA sheared and 20nt-size-selected before cDNA synthesis. The within-lab correlations are best, especially those performed most recently (Singh vs Fadda). The Manful dataset was generated using older library construction and sequencing technology. The magenta dashed line is perfect correlation.

A) Bloodstream forms, Manful vs. Fadda, rRNA-depleted RNA.

B) Bloodstream forms, Singh poly(A)+ vs. Manful poly(A)+

C) Procyclic forms, Singh poly(A)+ vs. Fadda rRNA-depleted

D) Bloodstream forms, Singh poly(A)+ vs. Vasquez poly(A)+

E) Procyclic forms, Fadda rRNA-depleted vs. Vasquez poly(A)+

F) Bloodstream forms, Fadda rRNA-depleted vs. Vasquez poly(A)+

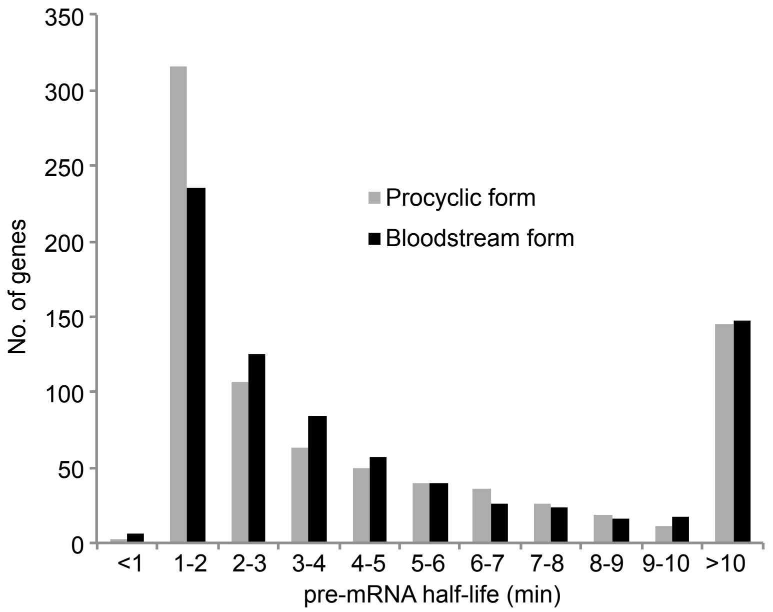

Supplementary Figure S3
Half-lives of pre-mRNAs
Precursor half-lives were placed into categories as indicated, and the number of mRNAs in each category was counted. For mRNAs with more than one annotated splice site, results are shown only for the site with the shortest half-life.

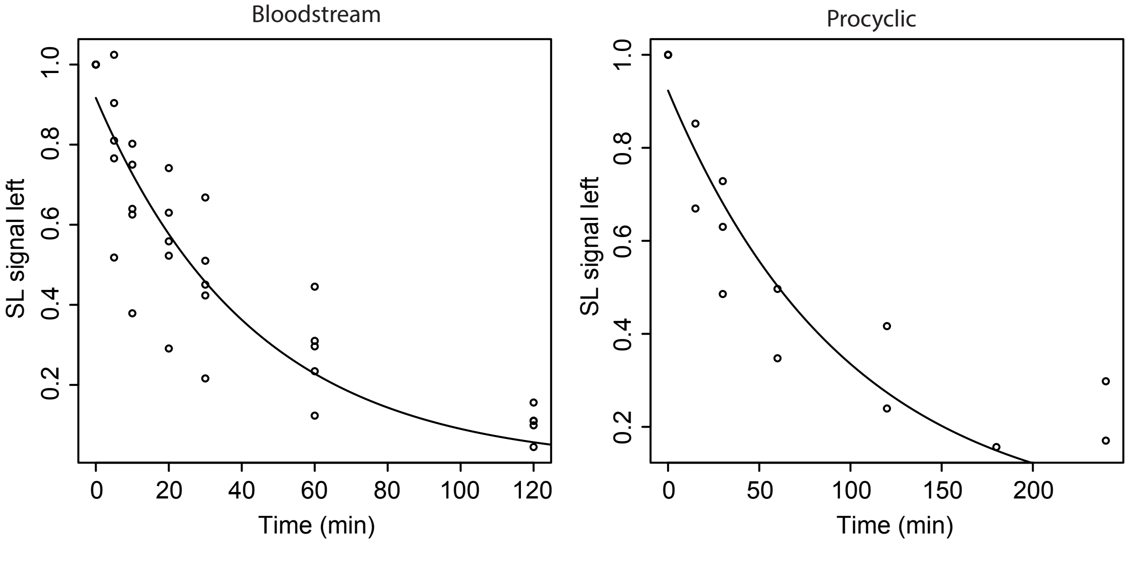

Supplementary Figure S4
SL signal calibration
Overall mRNA decay was determined by fitting an exponential curve into data points of 3 biological replicates (and 2 technical replicates for BF) of the Northern SL hybridization blots. The values used to normalize the RNAseq data corresponding to the curve are: bloodstream forms t0 0.92, t5 0.82, t10 0.73, t20 0.59, t30 0.46, t60 0.23, t120 0.06. procyclic forms t0 0.92, t15 0.79, t30 0.68, t60 0.50, t120 0.27, t180 0.15, t240 0.08.

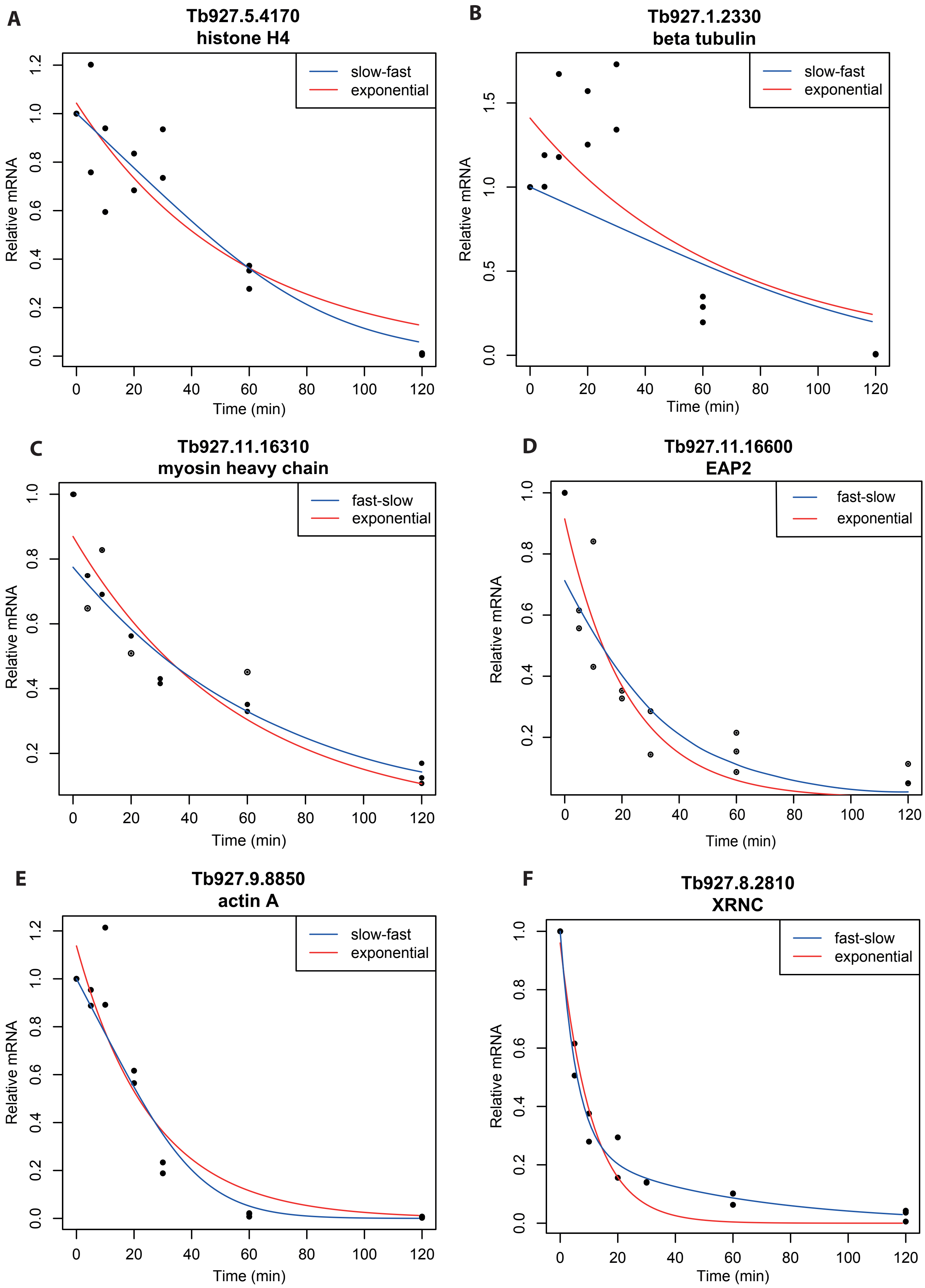

Supplementary Figure S5: Curve-fitting for selected genes
Individual relative mRNA levels are shown for the different time points. Different curves were fitted as indicated. In each case, the exponential pattern was rejected in favour of a slow-fast or fast-slow model. Results are for:
A) Tb927.5.4170; B) Tb927.11.16310; C) Tb927.1.2330; D) Tb927.11.16600; E) Tb927.9.8850; F) Tb927.8.2810
Note that the data for E and F are also shown In Figure 2; we show them here as well because it is easier to compare the two curves. without the Northern and qPCR results.

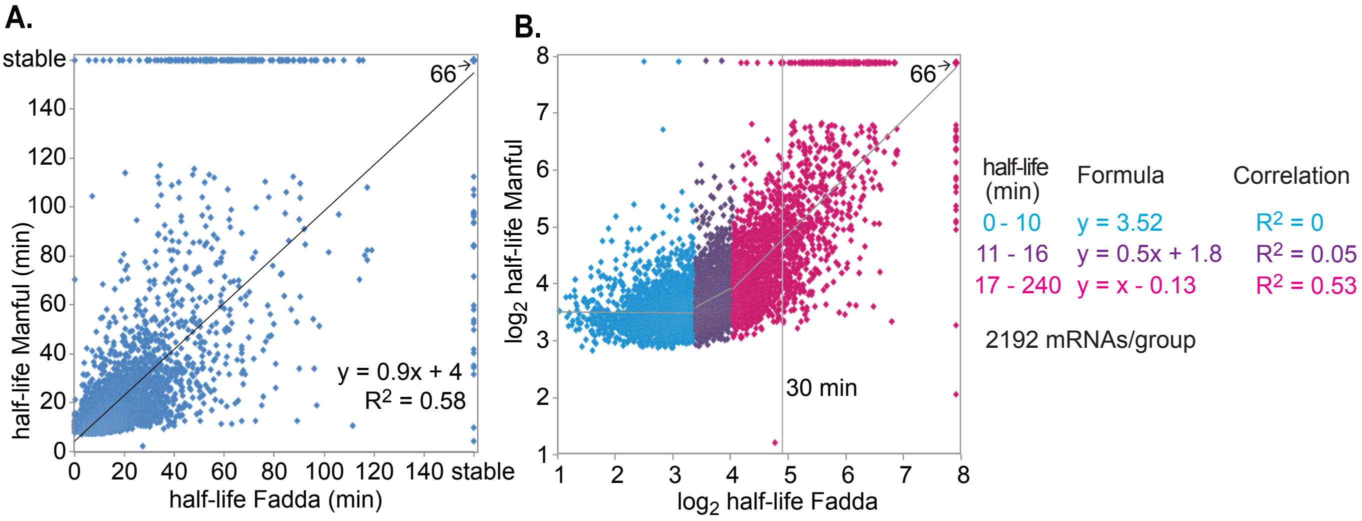

Supplementary Figure S6

Comparison of the current half-life results with previous ones.

A. The half-lives obtained for bloodstream-form mRNAs in this paper are compared with those calculated in Manful et al., (Manful et al., 2011), from incubation with Actinomycin D for 30-min. Stable mRNAs (half-life >120 min) were arbitrarily assigned a half-life of 240 min. Results are shown on a linear scale. The P value from a Students t-test (comparing the two datasets and counting only half-lives between 2 min and 120 min) was 1.4 E-37.

B. Same results as in (A), but all data are log2 transformed and the formulae and correlation coefficients are for the log-transformed data. The mRNAs are divided into three equally-sized groups according to half-life. The correlation is best for more long-lived mRNAs. This may be partly because the half-lives are nearer to the 30-min time point used in the Manful study. The very short-lived mRNAs yield very few read counts at the 30-min time-point, which makes half-life estimates based on that point alone inaccurate: note that no half-lives lower than 7 min could be measured.

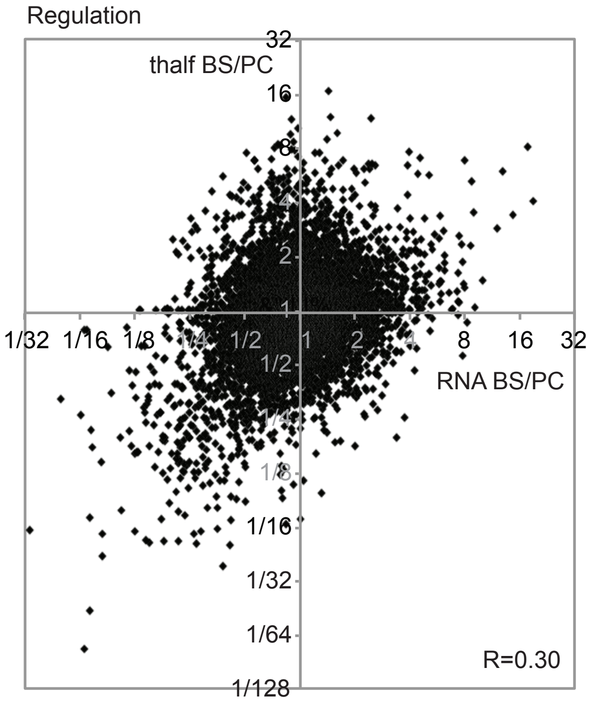

Supplementary Figure S8

Regulation of abundance correlates poorly with regulation of half-life.

The steady-state mRNA abundance in bloodstream forms, divided by that in procyclic forms, is on the x-axis, while the corresponding half-life ratio is on the y-axis.

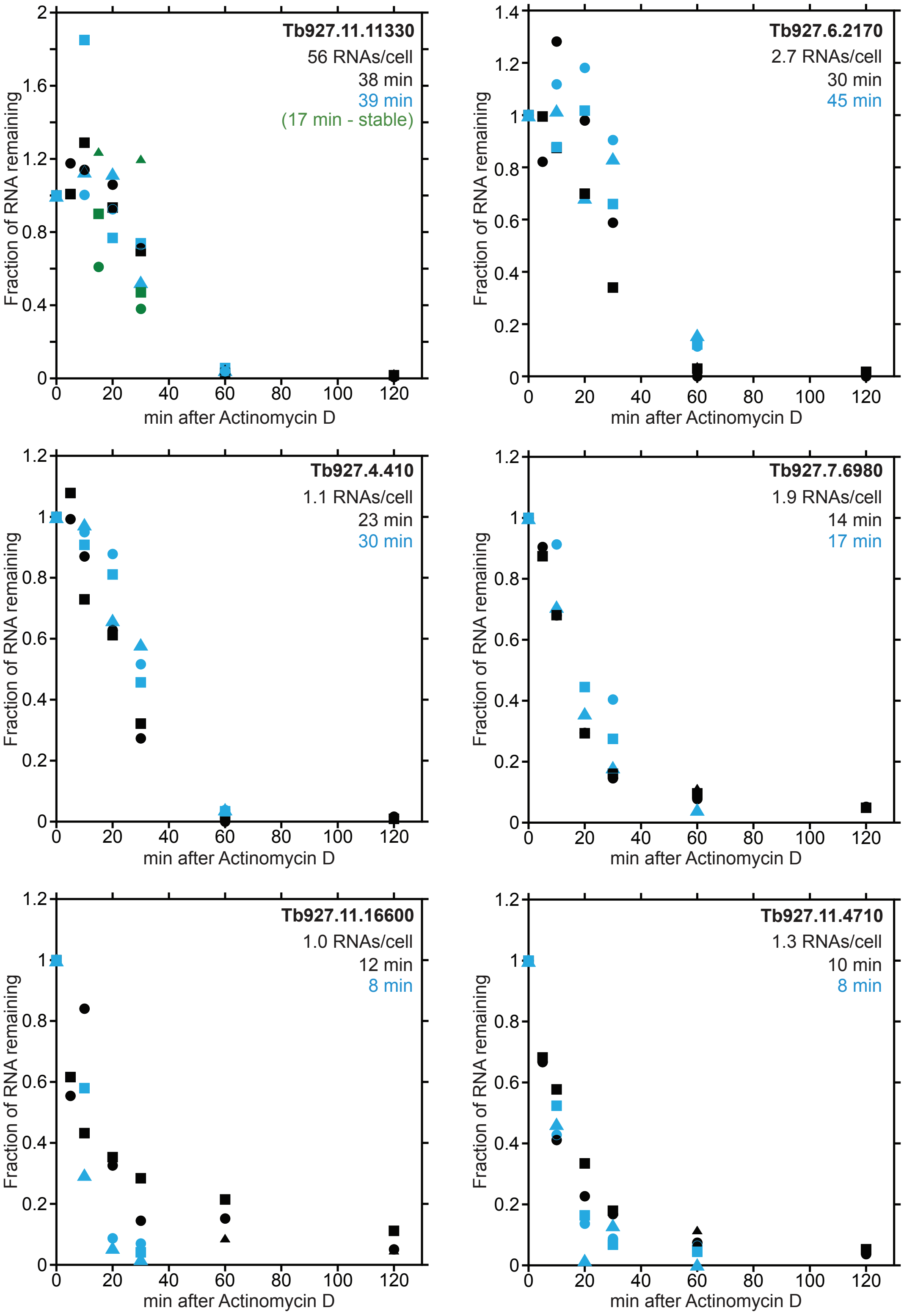

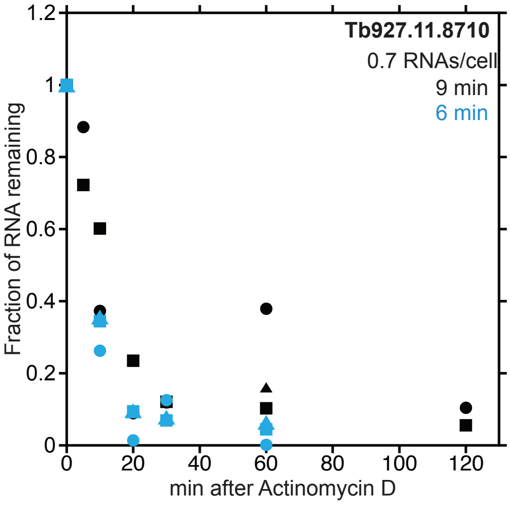

KEY RNASeq
q-RT-PCR
Northerrn blot

Supplementary Figure S7 part 1
Comparison of RNAseq degradation curves with published Northern blot or qRT-PCR measurements. RNASeq results are in black, Northern results in green and real-time qRT-PCR results are in cyan. The Northern and real-time qRT-PCR PCR data are those that were used to calculate half-lives listed in Table 2. The different symbols represent different biological replicates for qRT-PCR. For Northerns, each symbol represents the average of at least 3 independent biological replicates, and the different symbols are from different replicate sets. For consistency, clear outliers in both datasets have been included. All results are for bloodstream forms.

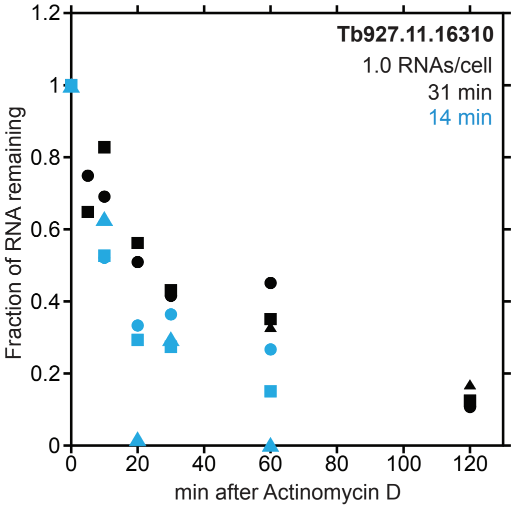

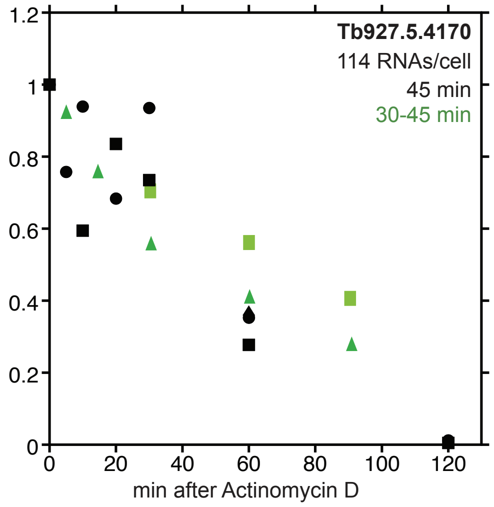

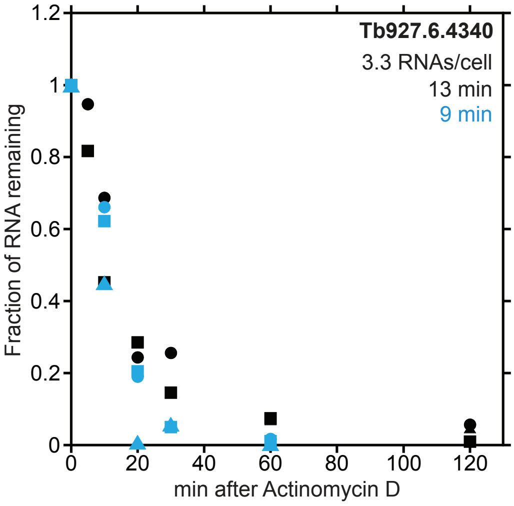

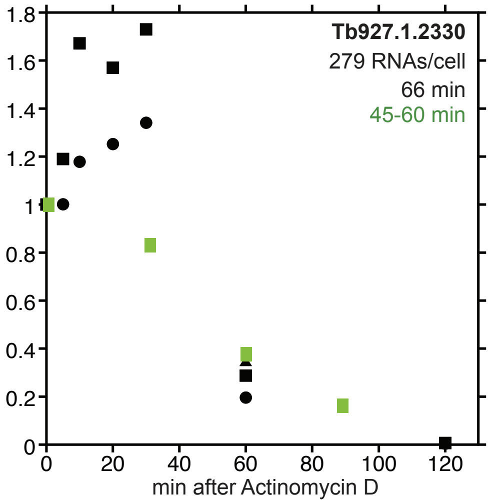

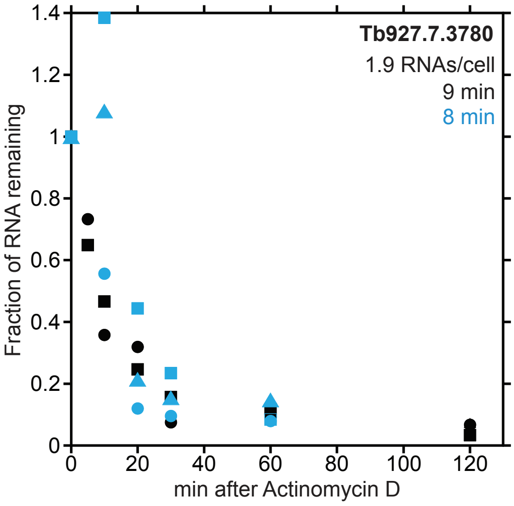

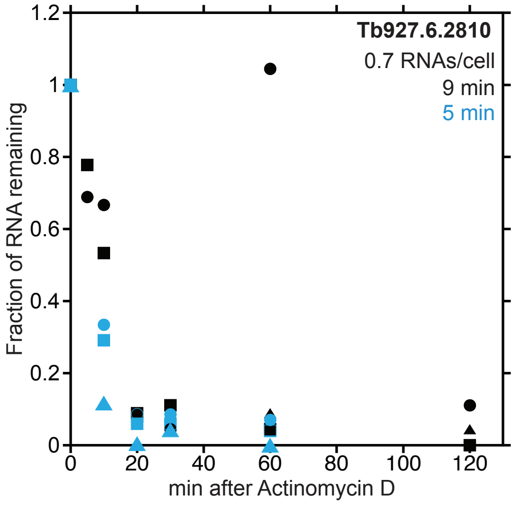

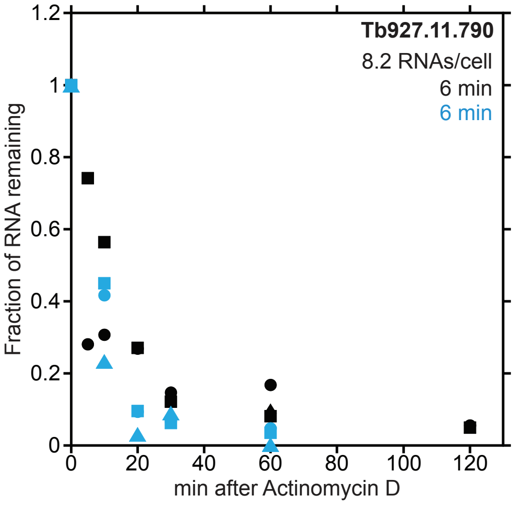

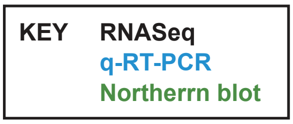

Supplementary Figure S7 part 2
Comparison of RNAseq degradation curves with published Northern blot or qRT-PCR measurements. RNASeq results are in black, Northern results in green and real-time qRT-PCR results are in cyan. The Northern and real-time qRT-PCR PCR data are those that were used to calculate half-lives listed in Table 2. The different symbols represent different biological replicates for qRT-PCR. For Northerns, each symbol represents the average of at least 3 independent biological replicates, and the different symbols are from different replicate sets. For consistency, clear outliers in both datasets have been included. All results are for bloodstream forms.

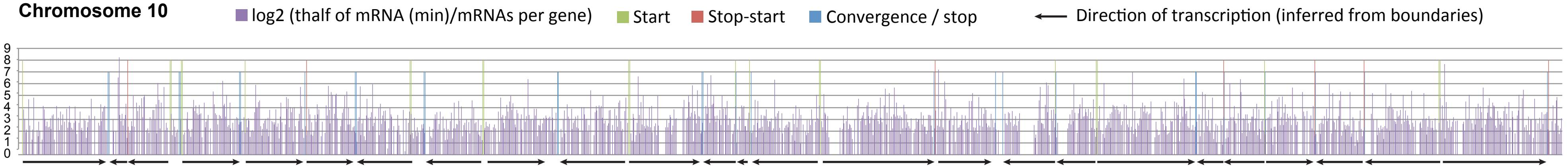

Supplementary Figure S9: The half-life - abundance ratio does not depend on chromosomal position

The Figure is a schematic depiction of chromosome 10. Genes were ordered according to their position on the chromosome. Transcription initiation (green and red bars) and termination points (blue bars) were assigned manually according to ORF direction and chromatin marks (Siegel et al., 2009). The arrows show transcription direction and were also placed manually, and since some transcription units are short there may be some errors in the assignments. Purple bars: the half-life (in min) was divided by the number of mRNAs/cell/gene, and the result was log2 transformed. High bars therefore mean that the mRNA has unexpectedly low abundance for its half-life. A space means only that there is no data for this particular position, or that it was not in the unique gene set.

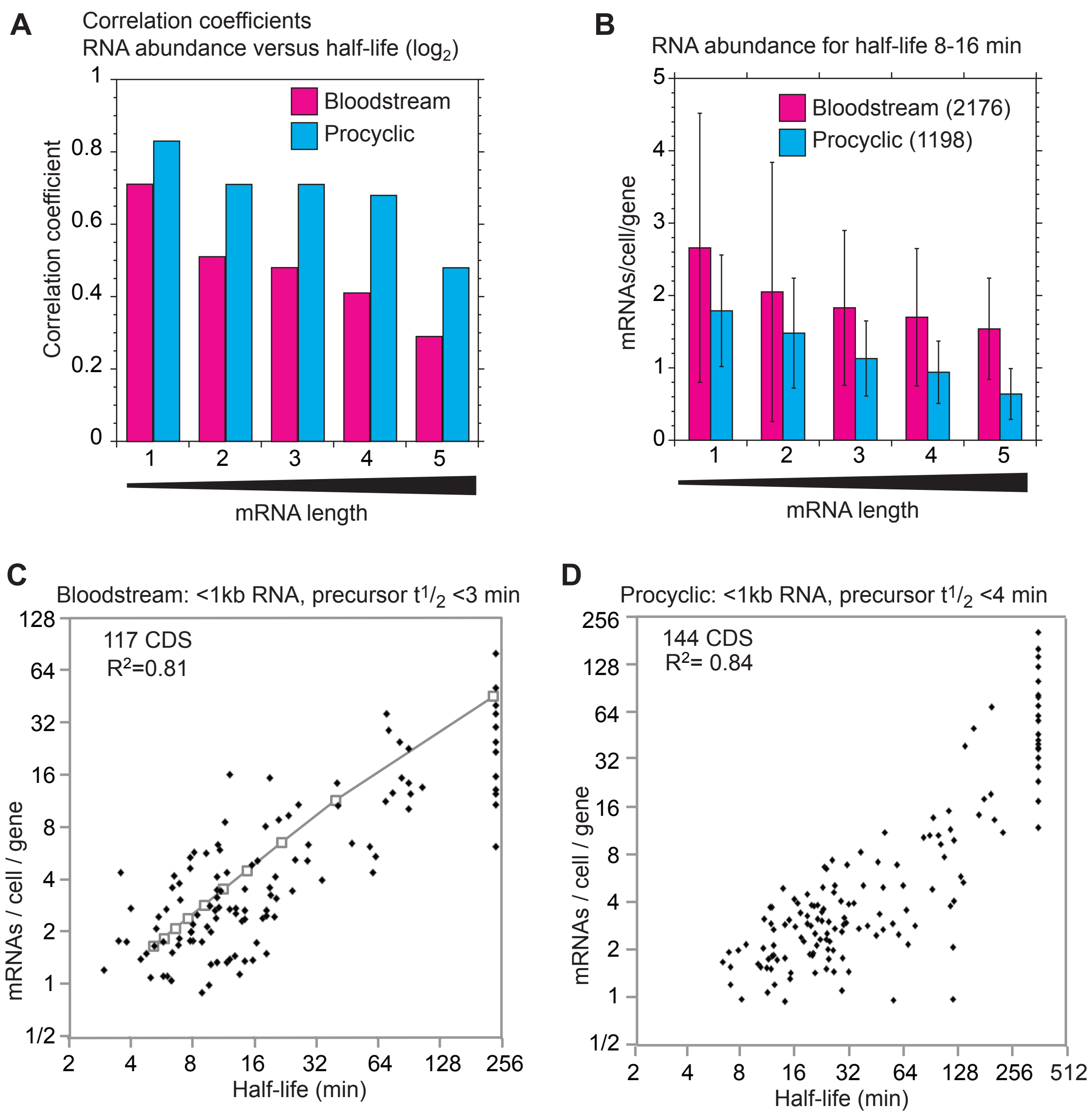

Supplementary Figure S10

The relationship between half-life and abundance

A. The mRNA lengths were extracted from TritrypDB and mRNAs lacking annotated splice or poly(A) sites were excluded. The coding sequences were then ranked according to the length and divided into 5 bins of equal size. Correlation coefficients were calculated, using log-transformed data, between the half-life and the number of mRNAs per cell per gene.

B. All mRNAs with half-lives between 8 and 16 minutes were extracted, ranked according to the length then divided into 5 bins of equal size (237 for procyclic forms, 435 for bloodstream forms). For each bin, the arithmetic mean of the abundance and the standard deviation are plotted. Note that given the sample size, standard errors would be too small to be visible on the plot. The mRNA abundances for each of bins 2-5 were significantly lower than those of bin 1 (Student T-test, P value < 0.02). Analyses using log-transformed data confirmed the relationship between length and abundance (not shown).

C. The number of mRNAs per cell per gene was plotted against the half-life for bloodstream forms, considering mRNAs that were less than 1kb long and had measured 5' precursor half-lives of less than 3 min. The grey squares indicate the results of modelling steady-state mRNA abundance with the published model (Haanstra et al., 2008) assuming a 5'-trans splicing half-time of 1 min. Note that the relatively high correlation coefficient for the data is partly caused by the fact that the sample size is smaller than in other plots.

D. The number of mRNAs per cell per gene was plotted against the half-life for procyclic forms, considering only mRNAs that were less than 1kb long and had measured 5' precursor half-lives of less than 4 min.

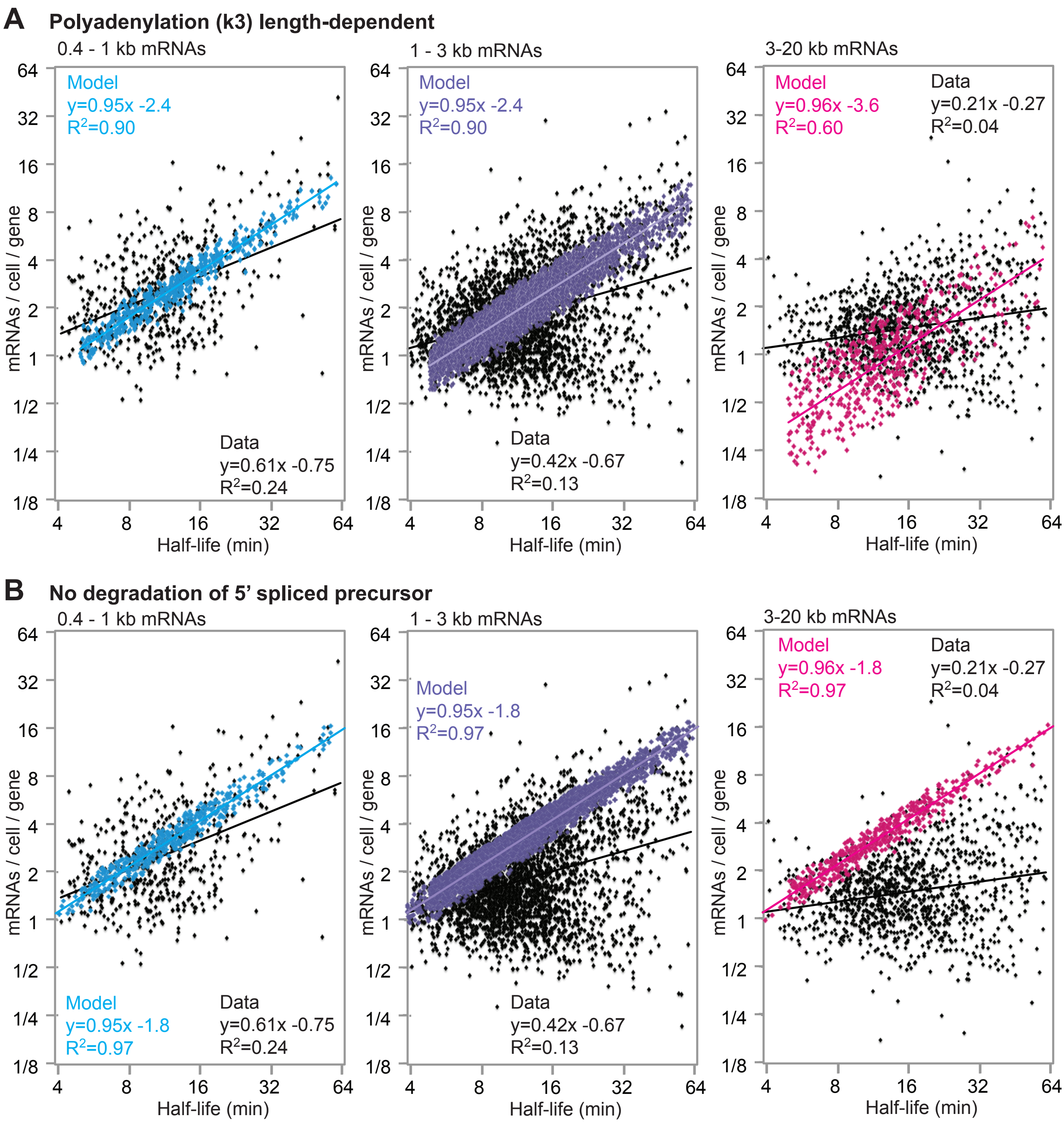

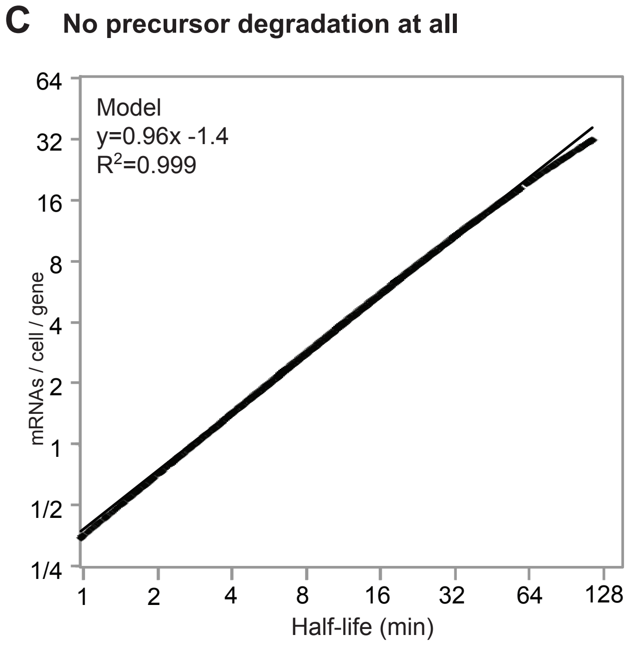

Supplementary Figure S11

Effects of changing the model parameters

Results are displayed as for Figure 7, panels C-E. Transcript length, splicing half-life and half-life of the mature mRNA were sampled as for the results in Fig 7.

A. The constant for polyadenylation (k_3; 0.41 min^{-1}) was multiplied by (600nt)/(mRNA length in nt) and degradation of the 5'-trans spliced precursor (k_5) was kept constant at 0.08 min^{-1}.

B. The constant for polyadenylation (k_3; 0.41 min^{-1}) was multiplied by (600nt)/(mRNA length in nt) and degradation of the 5'-trans spliced precursor (reaction 5) was removed.

C. Similar to (B), but reaction 4 (degradation of the un-spliced precursor) was also removed
